# Hydrothermal activity, functional diversity and chemoautotrophy are major drivers of seafloor carbon cycling

**DOI:** 10.1038/s41598-017-12291-w

**Published:** 2017-09-20

**Authors:** James B. Bell, Clare Woulds, Dick van Oevelen

**Affiliations:** 1 School of Geography, University of Leeds, Leeds, LS2 9JT UK; 2 Life Sciences, Natural History Museum, Cromwell Rd, London, SW7 5BD UK; 3 Centre for Environment, Fisheries and Aquaculture Science, Lowestoft, NR33 0HT UK; 4 Department of Estuarine and Delta Systems, Royal Netherlands Institute for Sea Research (NIOZ) and Utrecht University, Yerseke, The Netherlands

## Abstract

Hydrothermal vents are highly dynamic ecosystems and are unusually energy rich in the deep-sea. *In situ* hydrothermal-based productivity combined with sinking photosynthetic organic matter in a soft-sediment setting creates geochemically diverse environments, which remain poorly studied. Here, we use comprehensive set of new and existing field observations to develop a quantitative ecosystem model of a deep-sea chemosynthetic ecosystem from the most southerly hydrothermal vent system known. We find evidence of chemosynthetic production supplementing the metazoan food web both at vent sites and elsewhere in the Bransfield Strait. Endosymbiont-bearing fauna were very important in supporting the transfer of chemosynthetic carbon into the food web, particularly to higher trophic levels. Chemosynthetic production occurred at all sites to varying degrees but was generally only a small component of the total organic matter inputs to the food web, even in the most hydrothermally active areas, owing in part to a low and patchy density of vent-endemic fauna. Differences between relative abundance of faunal functional groups, resulting from environmental variability, were clear drivers of differences in biogeochemical cycling and resulted in substantially different carbon processing patterns between habitats.

## Introduction

## Sedimented Hydrothermal Vents

Following the discovery of hydrothermal vents on the East Pacific Rise in the late 1970s, it has become clear that chemosynthesis represents a vital carbon fixation pathway in the deep-sea^1,2^, supporting a unique diversity of fauna^3^. Hydrothermal vents have a diverse range of geological and geochemical drivers, resulting in several distinct types in soft and hard substratum settings but so far, research has focussed upon hard substratum vent systems, since they are undoubtedly the majority of systems. Sedimented hydrothermal vents (SHVs) are those where hydrothermal fluid vents through soft-sediment and, like their hard substrate counterparts, are enriched with certain chemicals, several of which may support chemosynthetic activity^4^. They have been discovered in diverse geological settings, both on the periphery of high-temperature vents^5–9^, and as independent environments^10–12^, meaning that they are widely distributed throughout areas with sufficient sediment flux and a potentially important source of food to deep-sea fauna, particularly those not endemic to hydrothermal vents.

Food webs at SHVs are supported by *in situ* chemosynthetic-based productivity and surface-derived organic matter and the soft-sediment setting allows for colonisation by both vent-endemic fauna and typical deep-sea soft-sediment fauna^4,6,8,13,14^. The combination of organic matter sources and differences in relative abundance of faunal functional groups, between sites of variable hydrothermal activity, create potential for a wide range of possible trophic activities. In such a complex setting it is challenging to determine the contribution of the various food sources and differences relating to hydrothermal activity based on empirical observations alone. These deep-sea ecosystems, owing to their extreme isolation, are some of the most poorly studied areas on the planet and the present study represents the first attempt to quantitatively describe seafloor carbon cycling in these settings. The model presented here provides a means to compare and contrast energy flows between and within physical and biotic carbon stocks and is particularly useful for datasets with significant uncertainty about parameters. The model is designed to support interpretation of the significance of chemosynthetic primary production to the resident (specialist and non-specialist) fauna in SHVs.

Owing to thermal decomposition of sediments, SHVs may contain high concentrations of chemosynthetic substrates, particularly methane^9,15,16^. Chemosynthetic production occurs through several pathways, such as sulphide or hydrogen oxidation or anaerobic oxidation of methane and there is now also evidence of diverse microbial lineages associated with other pathways, such as iron oxidation^17–20^. Fauna living in these habitats have access to both chemosynthetic and photosynthetic energy sources, while their distribution and behaviour reflect a balance between their ability to tolerate the high temperature, reducing conditions introduced by the influx of hydrothermal fluid and selectivity of the relative amounts of organic matter present^4,13^. The environmental gradients between SHVs and background sediments therefore create a range of assemblage compositions, trophic structures and pathways for *in situ* productivity^4,6,13,17,21,22^. We report here, for the first time in a sedimented vent system, how this affects energy transfers within food webs and the extent to which chemosynthetic production subsidises the diet of non-specialist fauna.

## Measuring food web interactions

Stable isotopes have been used widely to study chemosynthetic activity in deep-sea food webs, but their interpretation is often complicated by variability and uncertainty in factors such as trophic discrimination factors and the range of OM sources available^23,24^. Owing to the small number of SHVs^6,12,25,26^ that have been studied and the multiple scales of variability within each site, quantitative estimates of the contribution of chemosynthetic OM to the faunal food web are lacking. Stable isotope mixing models have been implemented for some Arctic SHVs^6^, which found wide ranges of chemosynthetic organic matter contribution to different taxa but generally, the lack of data (e.g. for trophic discrimination factors) has precluded the use of these tools to quantify trophic interactions and biogeochemical cycling^27^. In this study, we take advantage of an extensive, multidisciplinary dataset available for the sedimented vent system at Hook Ridge in the Bransfield Strait (62°S), in the Southern Ocean, which hosts the most southerly known vent field worldwide^28^. These data include macrofaunal composition and stable isotopic signatures; bacterial fatty acids; particulate organic carbon flux; temperature; flux of chemosynthetic substrates and a range of metabolic constraints. The Bransfield SHV complex is one of the first such systems to have a sufficient breadth of the requisite data inputs necessary to apply such an approach. Several other deep-sea systems have been studied in this way^29–31^ but previous applications of this technique have not considered the role of primary productivity in the deep-sea.

We designed three linear inverse models (LIMs), one each for two sites along a gradient of variable hydrothermal activity (low activity: Hook Ridge 1 [~24 °C] and high activity: Hook Ridge 2 [~48 °C]) and a non-vent site (Bransfield Off-Vent [~−1 °C]) to quantify and contrast carbon flows in the benthic food web. LIMs are powerful tools to incorporate multi-disciplinary datasets, provided that they can be expressed in a common currency (in this case, carbon), to address questions regarding food web complexity and biogeochemical cycling. LIMs rely upon a series of constraints upon flows and stocks (e.g. growth rates or biomass) in order to reconstruct likely flow rates between stocks, at a level of detail that would be impossible to achieve through field measurements. Literature data are not implemented as ‘hard’ and fixed values, but rather as ‘soft’ ranges, to account for the fact that some types of information are better constrained than others^32^. By resolving the field observations and literature data sources simultaneously, the ‘best’ carbon flux estimates and their associated uncertainty are estimated.

Macrofauna with endosymbionts were observed at both the vent sites (*Sclerolinum contortum*) and non-vent sites (*Siboglinum* spp.)^13^. LIMs are especially suited for environments where repeated or detailed measurements are not practical (e.g. the deep-sea) and represent a powerful tool to address uncertainty in these settings. A comprehensive dataset was implemented in the LIMs including: organic and inorganic geochemistry^10,11,13,17,33^; microbial composition and stable isotopic signatures^17^; faunal composition and stable isotopic signatures^13,17^; sediment community respiration rates as well as more general data for the Bransfield Strait and other deep-sea food webs^29,34–37^. Here we apply a LIM approach for two sites of variable hydrothermal activity and one off-vent site to address the following hypotheses that hydrothermal activity: 1) supports *in situ* production that subsidises the diet of local fauna; 2) creates structural differences between food-web networks and 3) increases faunal trophic diversity.

## Results

The following results are based on comparisons of flow values produced by each model. Each model had the same basic structure of possible flows but parameter and stock size values varied between sites (see methods section for further details). These differences are ultimately responsible for variability between each site. Data presented are mean values (±1 standard deviation) from 100 000 iterations for each model.

### Contribution of Chemosynthetic OM to the food web

**Figure 1:**
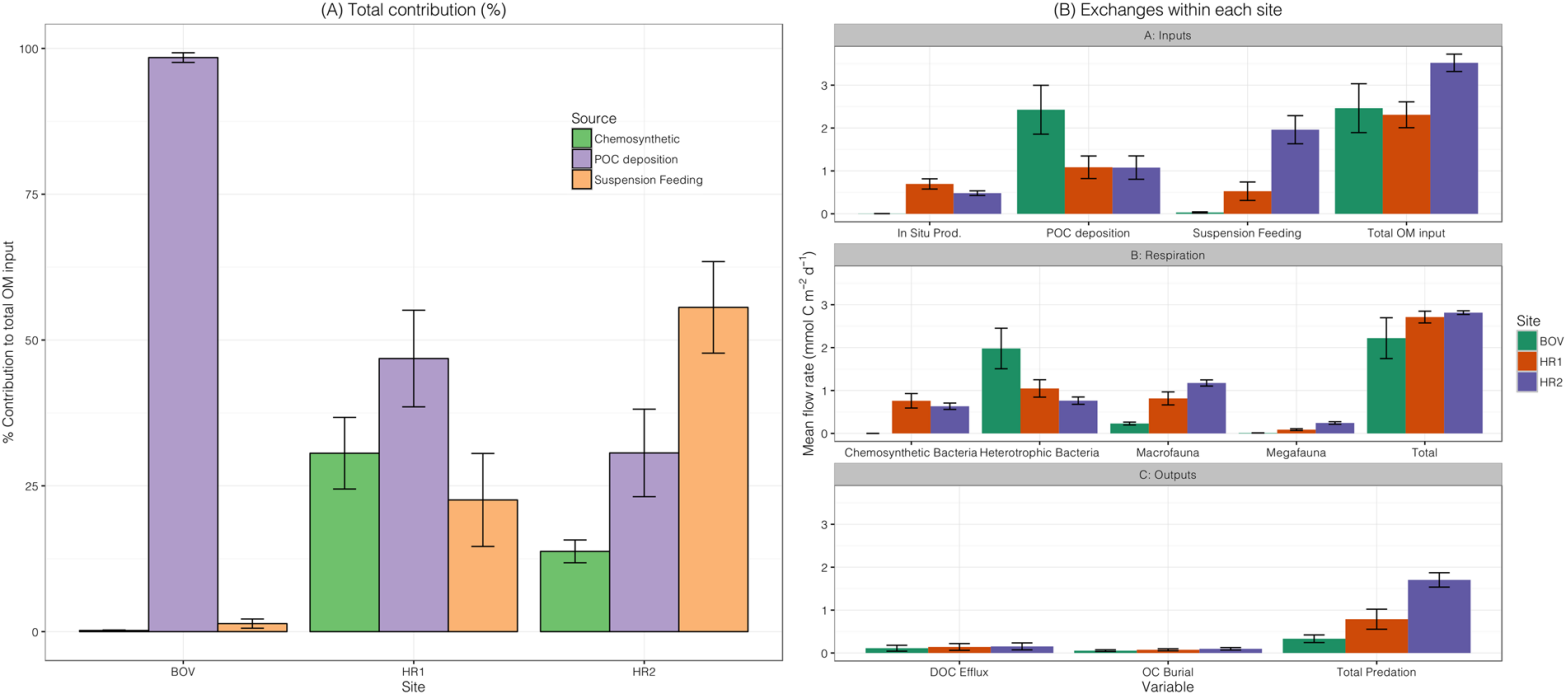
(**A**) Percentage contribution of OM inputs at each site (±1 S.D.); (**B**) Comparison of selected variables of external and internal cycling values (±1 S. D.). POC = Particulate organic carbon. DOC = Dissolved organic carbon. OC = Organic carbon. *In situ* production, suspension feeding and total OM inputs are given as net values (i.e. corrected for metabolic constraints of the relevant taxa).

Chemosynthetic OM constituted 0.2% (±0.07) of the net total OM inputs at the off-vent site, compared with 30.6% (±7.97) at Hook Ridge 1 (HR1) and 13.8% (±1.95) at Hook Ridge 2 (HR2) (Fig. 1A). The net total OM input to each site ranged between 2.31–3.52 mmol C m^−2^ d^−1^ and net contribution of in situ chemosynthetic primary production varied substantially between sites (Fig. 1; Table 1). Other OM inputs were direct POC deposition and inputs from suspension feeders. The models suggested that at the off-vent site, direct POC deposition was significantly higher than at either Hook Ridge 1 or 2 (Table 1) (97.4 and 97.5% of BOV solutions greater than Hook Ridge 1 & 2 respectively). Net suspension feeding was highest at Hook Ridge 2 (Table 1) and was the only site where suspension feeding represented the dominant input of OM. POC deposition was the dominant mode of OM delivery at BOV and HR1.

**Table 1:** Selected variables from each model (mean ± 95% confidence intervals). Net fluxes are corrected for relevant constraints (e.g. respiration or uptake efficiency), which also accounts for HR1 where total respiration is higher than the net OM inputs (because OM inputs are already adjusted for bacterial and metazoan respiration).

Variable	Carbon flux (mmol C m^−2^ d^−1^ ±S.D.)		
	BOV	HR1	HR2
*Inputs*			
POC deposition	2.43 (±0.57)	1.09 (±0.26)	1.08 (±0.27)
Net Suspension Feeding	0.03 (±<0.01)	0.53 (±0.22)	1.96 (±0.33)
Net Chemosynthesis	<0.01 (±<0.01)	0.70 (±0.12)	0.48 (±0.05)
Net Total OM Input	2.46 (±0.57)	2.31 (±0.30)	3.52 (±0.20)
Gross Total OM Input	2.72 (±0.54)	3.72 (±0.30)	4.77 (±0.20)
*Internal C Processing*			
Faunal Detritus Production	4.02 (±0.96)	3.54 (±0.57)	3.08 (±0.20)
Total C Respiration	2.21 (±0.48)	2.71 ( ± 0.14)	2.82 (±0.04)
*Outputs*			
Burial of Organic C	0.05 (±0.01)	0.08 (±0.02)	0.10 (±0.03)
DOC Efflux	0.11 (±0.07)	0.14 (±0.08)	0.15 (±0.08)
External Predation	0.33 (±0.09)	0.79 (±0.23)	1.70 (±0.17)

Respiration was the dominant fate for organic carbon in each model (Table 1), accounting for 59.0–81.7% of total OM input at each site, though there were clear differences in the proportion accounted for by each compartment. Mean total respiration was slightly higher at the vent sites than at the off-vent site (Table 1; Fig. 1B). Heterotrophic bacterial respiration accounted for 89.2% of the total at the off-vent site but only 49.6–66.7% at the vent sites. Macrofaunal respiration accounted for 30.1 and 41.8% of total respiration at HR1 and HR2 respectively, compared with 10.4% at the off-vent site. Megafaunal respiratory demands were comparatively limited (0.4–8.6% of total at each site). Respiration represented a loss of C from the system as DIC but 39.6–74.1% of this was recycled as *in situ* production at the vent sites. Detritus production (by all microbial and faunal compartments) exceeded total input of allochthonous POC (direct input plus net suspension feeding) at both BOV and HR1 (pairwise comparisons: >99.9% of solutions greater for detritus production) but at HR2, faunal recycling rates were comparable to POC input (Table 1) (Means 3.07 and 3.04 mmol C m^−2^ d^−1^ respectively).

**Figure 2:**
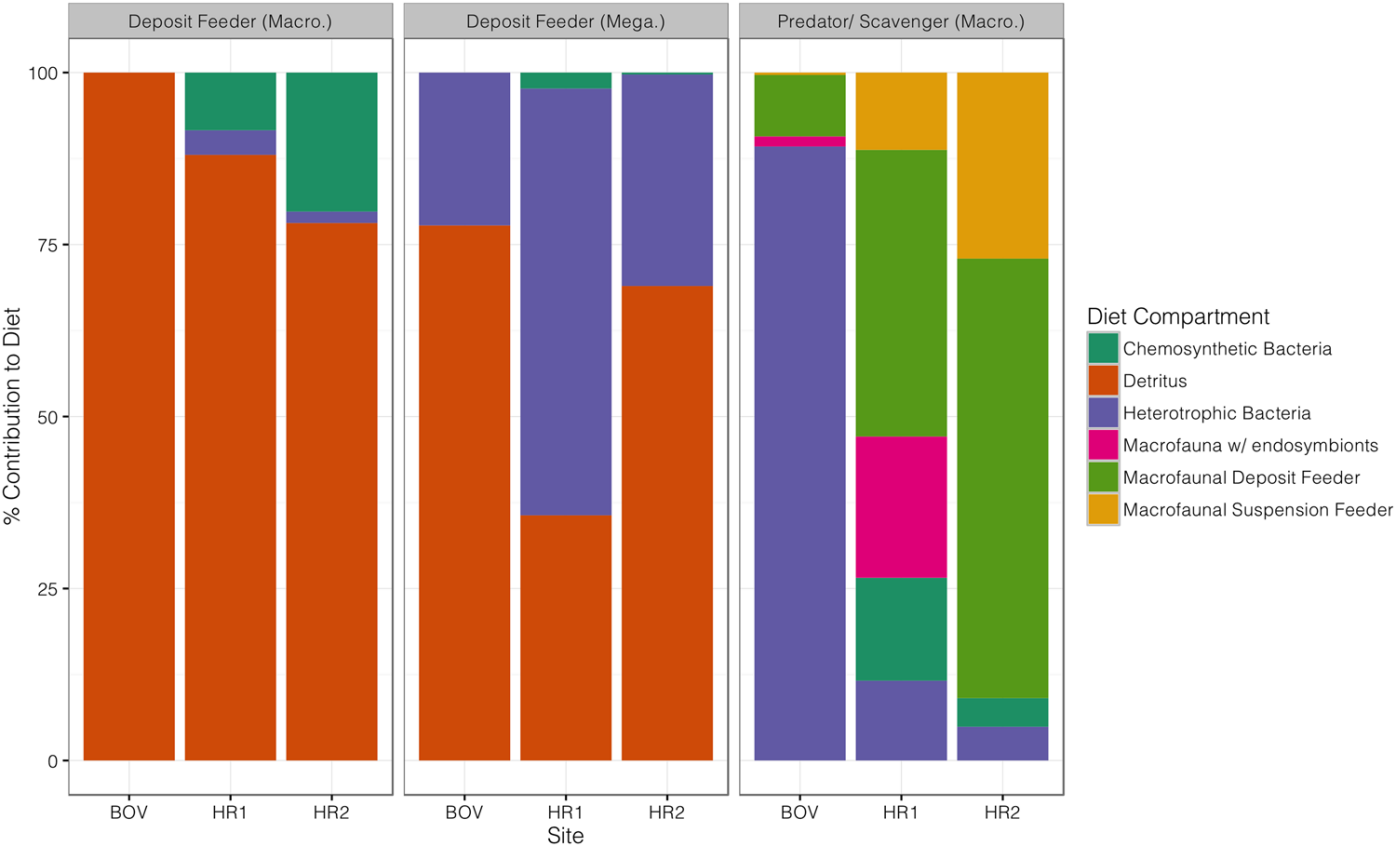
Percentage diet composition of deposit feeders and predators/scavengers at each site, along a gradient of hydrothermal activity. Macro. = Macrofauna; Mega. = Megafauna.

Several compartments had their dietary composition fixed by the model structure (e.g. Endosymbiont-bearing siboglinids could only receive organic matter via in situ chemosynthetic OM fixation owing to their lack of feeding organs) but others were able to vary their diet according to isotopic variability and stock size. Diet composition varied between sites particularly for predators/scavengers (Fig. 2) (Chi-sq: χ^2^ = 172.77; p < 0.001). Deposit feeder diet was generally dominated by detritus (35.6–100%; Fig. 2) but also included heterotrophic and chemosynthetic bacterial carbon in varying amounts (0–62.0% and 0–20.2% respectively). Predator/scavenging faunal diet composition was the most different between sites and included significant proportions of bacterial carbon (9.1–89.3% of diet; Fig. 2), overlapping with deposit feeder diets, particularly at BOV. Predator/scavenger diet at the vent sites was dominated by predation upon macrofaunal deposit feeders (41.7 and 63.9% of diet at HR1 and HR2 respectively), but was also comprised of significant amounts of other sources (e.g. macrofauna with endosymbionts at HR1; 20.5% of diet or suspension feeding macrofauna at HR2; 27.0% of diet; Fig. 2). Predator/scavengers at Hook Ridge 1 had the greatest trophic diversity and did not seem to be as dependent upon a single source to the same extent as at other sites. This suggests that trophic diversity amongst higher trophic level fauna at SHVs may be highest at intermediate levels of hydrothermal activity.

DOC efflux and organic carbon burial rates were broadly similar between all sites and the major C loss (other than respiration) was external predation by motile megafaunal predators (e.g. fish) that were not included within the model. Mean external predation rates were highest at HR2 and lowest at BOV (Table 1) and were significantly different between all sites (pairwise comparisons all >98% of solutions greater in one model).

### Food web structure

**Figure 3:**
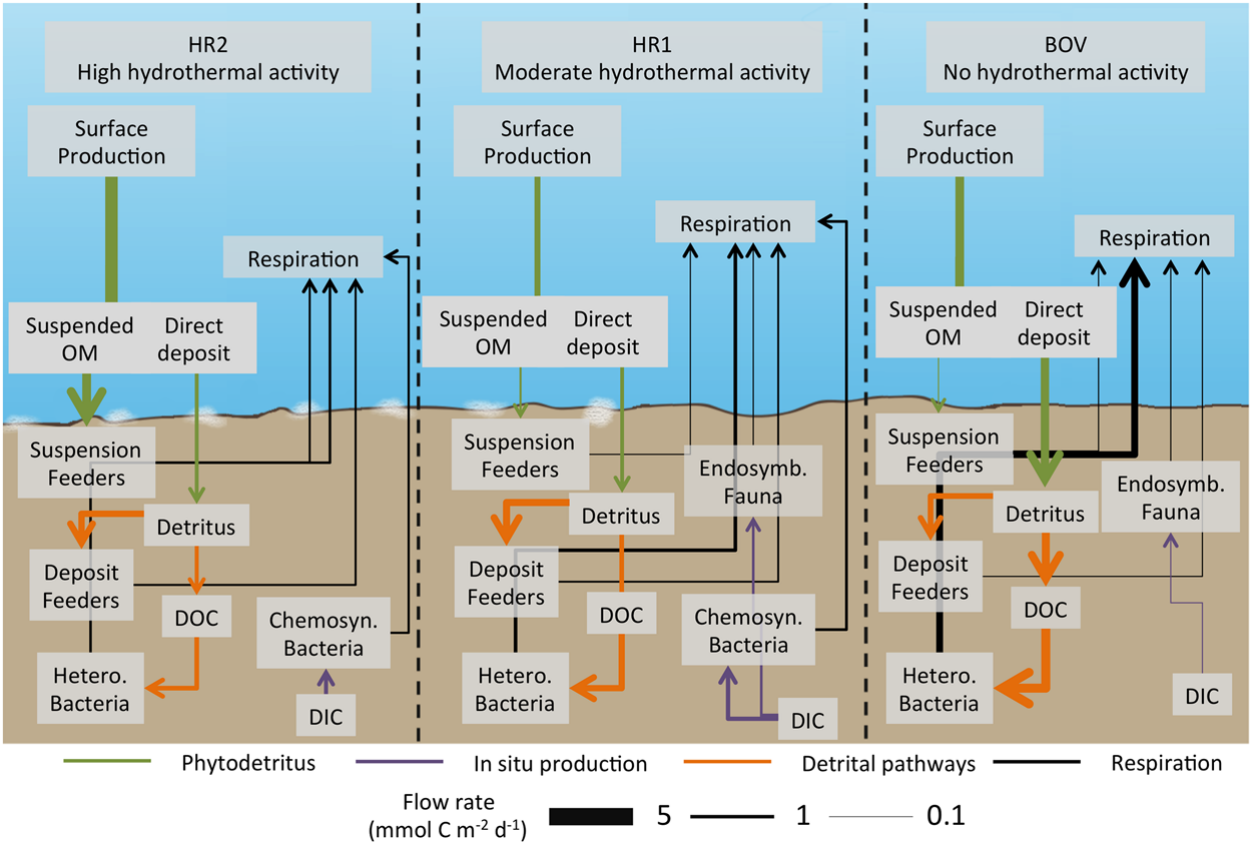
Selected mean carbon flows between food web compartments at each site. Arrow thickness = flow rate. White patches = bacterial mat. See supplementary figure for full details of exchanges between compartments.

Mean food web structures were different between each site, both in terms of the dominant flows and also the overall structure of the network (Fig. 3). Some of these disparities were because of differences in stock sizes of individual functional groups between sites (e.g. Macrofauna with endosymbionts absent from HR2), meaning that some flows were absent or differed in magnitude. Bacterial respiration was the dominant biological process (1.98 mmol C m^−2^ d^−1^ ± 0.47) at the non-hydrothermally influenced site (BOV) and, whilst it was still an active flow at HR1 and HR2 (1.05 mmol C m^−2^ d^−1^ ± 0.20 and 0.76 mmol C m^−2^ d^−1^ ±  < 0.09 respectively), other flow magnitudes were greater (e.g. macrofaunal deposit feeding at HR1 = 1.92 mmol C m^−2^ d^−1^ ± 0.51 or macrofaunal suspension feeding at HR2 = 2.57 mmol C m^−2^ d^−1^ ± 0.36).

### Network indices

Mean total system throughflow, the sum of all flows between compartments, was greater at the vent sites (12.70–13.03 mmol C m^−2^ d^−1^, compared with 11.56 mmol C m^−2^ d^−1^ at BOV) but the non-vent site had a higher proportion of recycled flows (Corrected Finn Cycling Index = 0.20 ± 0.06), as compared to HR1 and HR2 (0.19 ± 0.04 and 0.10 ± 0.01 respectively). BOV was the most strongly internally structured network (compartmentalisation index 0.33) though differences were generally small between all three sites (HR1: 0.32 & HR2: 0.30), indicating that the network was marginally more partitioned at the off-vent site. Average mutual information, a measure of the amount of constraint upon each flow, was lowest at the off-vent site (1.91 ± 0.06) and highest at HR1 (2.00 ± 0.03), suggesting that at the off-vent site, despite the higher degree of compartmentalisation, exchanges between stocks were more flexible.

## Discussion

**Figure 4:**
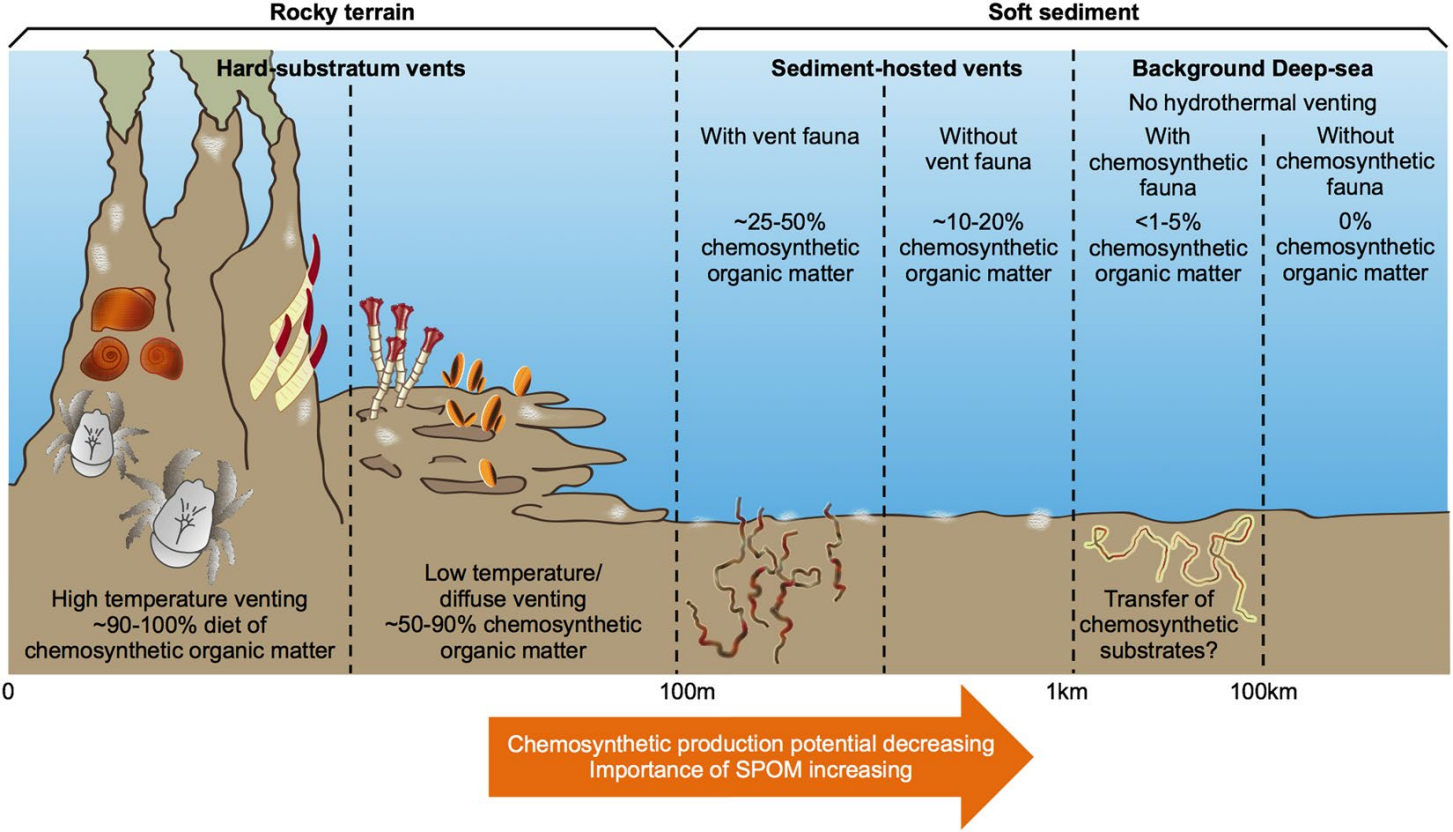
Differences in the potential contribution of chemosynthetic organic matter to different hydrothermal vent types, with representative taxa included for reference. SPOM = Surface-derived Particulate Organic Matter. White patches = bacterial mats. Figure credit: Alison Manson, University of Leeds.

This study provides quantitative estimates of the contribution of chemosynthetic OM to the food web along a gradient of hydrothermal activity at SHVs. Estimated contributions of chemosynthetic OM are most consistent with the stock sizes of chemosynthetic primary producers at each site, ranging from 0.2–30.6% of net total OM inputs. This highlights the importance of chemosymbiotic metazoa in mediating carbon flows at hydrothermal vents and clearly demonstrates that variability over several spatial scales can result in significant differences in the importance of various OM sources, both at soft sediment and hard substratum systems^25,26,38^. This complexity is consistent with studies of other similar systems^8,9,12^ and complements findings from the Guaymas basin of compositional changes relating to chemosynthetic substrate availability^9^. In this case, linear inverse modelling has been used to extend the available observations to encompass a range of carbon stocks and estimate the exchange rates between them at a level of detail that would not be possible in the field. At Hook Ridge 1, where chemosynthetic production represented 30.6% of the total OM inputs, this resource seemed to be widely utilised. This was in part due to the increased availability, but also the wider diversity of the sources. Chemosynthetic bacterial mat was available at both Hook Ridge sites but at HR1, the vent-endemic *Sclerolinum contortum* provided an additional means to route in situ production directly into metazoan biomass, potentially providing trophic support to predatory/scavenging fauna that may not otherwise have been able to feed directly upon locally produced OM. Thus, it is clear that symbiont-hosting megafauna can provide an important means towards integrating chemosynthetic production into the wider food web, and subsidising the diet of non-specialist fauna. The sites described here represent a permutation of the conceptual model of a hydrothermal vent food web^39^, each with one or more of the basic functional groups missing. The present study provides a basis for estimating how reorganisation of the functional composition of a food web can restructure the energy flows between stocks and, when coupled with physico-chemical factors, may result in substantially different flow networks. This has clear relevance to trophodynamics in other marine chemosynthetic ecosystems and suggests that patch-scale abundance of key functional groups, like endosymbiont-bearing fauna, is likely to considerably influence the transfer of chemosynthetic carbon into seafloor food webs (Fig. 4).

At the off-vent site, net chemosynthetic production was very low (<0.01 mmol C m^−2^ d^−1^), owing to the small population sizes (64–159 ind. m^2^) of endosymbiont-bearing fauna (*Siboglinum* sp.) at these sites^13^. Chemosynthetic OM contributed the largest proportion of total OM inputs at Hook Ridge 1 (Fig. 1A), a site where dense (but patchy) populations of a vent endemic species (*Sclerolinum contortum*) was observed that fix inorganic carbon into organic matter in situ^13,17,40^. At Hook Ridge 2, chemosynthetic OM contribution was lower (13.8%), owing in part to the absence of populations of *Sclerolinum* sp. at this site. Chemosynthetic bacterial production was broadly similar at the vent sites (HR1: 0.50 ± 0.11 and HR2: 0.48 ± 0.05 mmol C m^−2^ d^−1^). Bacterial biomass at Hook Ridge 1 was also much higher than in non-vent areas^17^, indicating greater potential for bacterial activity at the vent sites. Bacterial biomass however is not necessarily a good proxy of activity, since bacterial populations can be relatively dormant and so biomass specific metabolic rates were not implemented in the model^41,42^.

Total OM input (chemosynthetic and surface-derived OM) was highest at HR2 (Fig. 1A) but the model results also indicated that direct POC deposition was highest at the off-vent site (Table 1). Available estimates of OM export from the surface ocean in the Bransfield Strait vary widely (0.7–27.1 mmol C m^−2^ d^−1^)^34,35,37^ and the model predictions are comparatively consistent between sites. These estimates are likely to represent a time-averaged signal as they are based on the amount of surface-derived OM required to support the observed stock sizes, which turn over at lower rates than the sub-annual variability in export production. POC flux at the vent sites was similar but only ~45% of that of the off-vent site, which seems unlikely. The spatial separation between sites is likely too small (~100 km between BOV and Hook Ridge) to account for these differences in direct POC flux. This suggests a model artefact, probably resulting from either the high uncertainty in POC flux estimates or the way in which the model partitions allochthonous inputs. At the vent sites, more surface-derived organic carbon was routed (rather than as direct POC deposition) through suspension feeding fauna, particularly at HR2 where they were relatively more abundant^13^. The off-vent site by comparison had a very small contribution of suspension feeding OM input, as these taxa were largely absent from the macrofauna at this site. Total allochthonous flux of OM (direct POC deposition plus removal of suspended POC through suspension feeding) was still different between sites, but the differences were less marked (range 1.62–3.04 mmol C m^−2^ d^−1^; Table 1) and had a much smaller range than the variability in POC data used to inform the model. We cannot provide confirmation of the differences in POC flux suggested by the models but if POC deposition was genuinely different between sites, a possible, though unconfirmed reason was that BOV, being situated towards the base of a continental slope, could have received additional input from material sliding down the basin margin. The vent sites were elevated above the surrounding seafloor so were less likely to receive additional OM inputs in this way^13^. It is also possible that the location of the vent sites on a mid-basin ridge, and the input of hydrothermal fluid, could have created local scale hydrodynamic conditions that influenced POC deposition rates. Differences in hydrodynamic regime between the basin axis and margins may also explain the relatively high abundance of suspension feeding fauna at HR2^13^.

Despite a lower overall faunal biomass at vent sites, particularly Hook Ridge 2^13,17^, these sites had higher mean total respiration than the off-vent site (Table 1). These differences were predominately a result of differences in temperature between sites (highest at Hook Ridge 2 ~48 °C^33^, compared with ~−1 °C at the off-vent site), meaning that total respiration was higher and faunal compartments accounted for a greater proportion of total respiration. We note that meiofaunal assemblages would likely have contributed to some of the observed differences in sediment community oxygen consumption and so the models probably over-estimated bacterial, macro- and megafaunal respiration rates. LIM models from the Hausgarten Observatory (2500 m depth, Arctic Ocean ~79°N, ~7 °E)^29^ indicated that meiofaunal respiration accounted for approximately 0.68% of total respiration, hence it is unlikely that meiofaunal respiration was sufficient to explain differences between sites. Respiration rates at Hausgarten were dominated by microbial activity (93%)^29^, similar to the off-vent site, where bacterial respiration accounted from 89.2% of mean total respiration (Table 1). This provides strong independent confirmation that the models presented here are behaving consistently with previous applications of this framework in other deep-sea ecosystems. Total bacterial respiration (heterotrophic and chemoautotrophic) rates were 1.98 (±0.47), 1.81 (±0.19) and 1.40 (±0.08) mmol C m^−2^ d^−1^ at BOV, HR1 and HR2 respectively, despite the higher bacterial biomass at the vent sites. Bacterial respiration accounted for just 66.7 and 49.6% at HR1 and HR2 respectively, with faunal respiration accounting for 30.0–41.8% of total respiration at the vent sites, compared with just 10.4% at BOV. Bacterial respiration and OM recycling are usually very dominant processes in non-hydrothermally influenced seafloor ecosystems^29,31^, often with little transfer of OM to metazoa^43^ but these models demonstrate that this is quickly overturned in higher energy systems in the deep-sea.

Network analyses indicated that the proportion of flows that were cycled within the food web was highest at the non-vent site (cFCI = 0.20), despite this site having the most compartmentalised network. Network compartmentalisation provides a means to investigate the extent to which in situ production was incorporated into the wider food web and was lowest at the most active vent site (HR2). This suggests that chemosynthetic carbon was generally well assimilated into the wider food web (rather than representing a spatially concurrent, yet isolated, set of trophic interactions). It also implies that the vent sites were generally more likely to be inhabited by more opportunistic fauna, with the off-vent site, being more stable, being inhabited by fauna with more finely partitioned niches, suggesting a pattern similar to that of declining food web complexity with increasing chemosynthetic substrate flux in seeps^8^. This corresponds to cycling efficiency, which declined with increasing hydrothermal activity, to 0.10 at HR2. The increase in temperature associated with higher hydrothermal advection caused an increase in bacterial and faunal respiration, resulting in a less efficient set of trophic linkages. It is also possible that the lower diversity at the most active vent sites meant that the food web was less efficient at processing the inputs of organic matter or that chemosynthetic OM was less efficiently recycled. Burial rates of organic carbon were higher at the vent sites but were not significantly different between sites (pairwise comparisons 11.6–27.8% smaller in one site), meaning that the differences in processing efficiency are most likely to be attributable to respiration differences.

Hydrothermal activity elicited considerable differences in diet amongst predator/scavengers (Fig. 2). At the off-vent site, predators predominately consumed bacteria and deposit feeders but also included a small contribution from endosymbiont-bearing fauna (1.5%). This is consistent with more detailed stable isotopic observations of a peracarid taxa (Neotanaidae) at this site, which had notably depleted carbon isotopic signatures, consistent with consumption of the methanotrophic *Siboglinum* sp.^17^. Additionally, many of the predatory polychaete fauna were small syllid polychaetes, which have been suggested to exhibit bacterivory^12,13,44^. However, the values associated with flow from bacterial carbon to predator/scavengers at the off-vent site had relatively high variability (0.20 mmol C m^−2^ d^−1^ ± 0.09), so these estimates should be treated with some caution. Predator/scavengers at Hook Ridge 1 had the most trophically diverse diet and this in part reflects the number of resources available (HR1 was the only site to include both chemosynthetic bacteria and endosymbiont-bearing fauna, as well as other macrofaunal functional groups). This contrasted with community bulk stable isotopic data^17^, where isotopic niche area (analogous to trophic diversity) was lower at the vent sites. The methane signature present in the off-vent siboglinids was apparently further offset in carbon isotopic signature from the other carbon sources available, and thus contributed to a larger isotopic niche area. Deposit feeders were the main source of food for predator/scavengers at both vent sites (41.7–63.9%, Fig. 2) and differences in the remainder of diet were generally consistent with stock sizes at each site (e.g. greater density of suspension feeders at HR2). Though we did not explicitly investigate predator trophic position, it is likely that the increased consumption of macrofaunal sources at vent sites would have resulted in a higher trophic position for predator/scavengers than at the off-vent site. This distinction is not clear from the bulk stable isotopic data^17^, perhaps because of confounding factors such as different nitrogen isotopic baselines between sites and unknown variability in trophic fractionation that can complicate interpretation of trophodynamics at hydrothermal vents. This demonstrates the value of the approach used here, as a means to circumvent the considerable uncertainty associated with deep-sea food webs.

The models presented here provide an important step towards understanding sedimented chemosynthetic ecosystems in the deep-sea. It remains challenging to use these results for an estimate of the contribution of SHVs to global benthic biogeochemical cycling, because the global extent of individual vent fields is largely unknown, both in the Bransfield Strait and in other systems in the Pacific, such as Middle Valley or Guaymas Basin. Additionally, some hydrothermal vent fields have peripheral areas of hydrothermal sediment^5,6,12^, generally of unknown size. The wide range of settings that could host SHVs indicates that their global extent may be significant and estimates from a large dataset of vent fields suggest that between 15 and 20% of all vent fields host some degree of sediment hydrothermalism. It is virtually impossible to provide a substantiated estimate of the importance of chemosynthetic activity in seafloor sediments without much more extensive data concerning the distribution of SHVs; the spatial extent of hydrothermal influence^45^ and an understanding of how representative the sites studied here are of other SHVs. This is a clear gap in current knowledge and we suggest that future hydrothermal vent research cruises could consider incorporating wider spatial coverage into their study design, to better capture this within-field variability and gradients of chemosynthetic OM utilisation (Fig. 4).

These results may also be useful to the study of other sedimented chemosynthetic ecosystems, such as cold seeps or mud volcanoes, or even to document trophic subsidies in shallow water commercial species^46^ as they suggest that in situ productivity can substantially reconfigure benthic carbon flows. *In situ* productivity and hydrothermal input were associated with clear differences in the relative abundance of faunal functional groups^13,17^ and carbon processing patterns (e.g. transfer pathways of OM into food web and differences in respiration), raising significant questions about benthic biogeochemical cycling. Faunal distribution is strongly partitioned according to species thermal niches^47^ and here we demonstrate that this could have major concomitant effects upon carbon processing patterns (Fig. 4).

The presence of chemosynthetic production at non-hydrothermally influenced sites raises important questions about the spatial scales over which chemosynthetic activity can contribute to energy flows within ecosystems^45^. Although the contribution was very small at the off-vent site (0.2% ± 0.07%; range = 0.03–0.72%), populations of *Siboglinum* sp. were much wider spread than populations of *Sclerolinum contortum* at Hook Ridge^13,17^. Samples for methane isotopic signatures were not available to this study but it worth noting that in the Bransfield Strait, *Siboglinum* sp. was found at all non-Hook Ridge sites^17^ and its carbon isotopic signatures (assuming little or no preservation effects) corresponded closely to that of thermogenic methane, previously observed at several sites throughout the basin^16^. The link between thermogenic sediment methane (apparently of hydrothermal origin) and chemosynthetic trophic support to non-vent ecosystems has not been made previously and, although unconfirmed, warrants further attention. Although these fauna generally occurred at low densities (25–135 ind. m^2^, compared with 32–4520 ind. m^2^ of *Sclerolinum contortum* at Hook Ridge^13^), their greater spatial extent could have resulted in comparable levels of total contribution of chemosynthetic carbon to the benthic food web across the Bransfield Strait. *Siboglinum* is a globally distributed genus and known from several contrasting systems^48–50^ and these results support the suggestion that it, and other similar taxa, may have far greater impacts upon benthic food webs throughout the deep-ocean than previously recognised, whether the methane fuelling the chemosynthesis is thermogenic or biogenic in origin.

## Conclusions

We have developed a quantitative ecosystem model of a sedimented vent system and show that the partitioning and processing of organic matter within the sediment is strongly influenced by the production and availability of chemosynthetic OM. We suggest that vent-endemic fauna are highly important for mediating transfer of local production into the food web, particularly for predatory or scavenging fauna. Contribution of chemosynthetic carbon was much more strongly influenced by the thermal niche of metazoan primary producers (not found at the more active site) than the levels of hydrothermal input and can vary substantially within vent fields. We also show that chemosynthetic production may have been much more widely available than previous recognised and suggest that it may influence benthic food webs over large areas.

## Materials and Methods

### Study site

The Bransfield Strait is a slow spreading basin (max. depth ~1900 m) located between the West Antarctic Peninsula and the South Shetland Islands (~62°20 S, 57°00 W). Along the basin axis there are three raised volcanic edifices (~1000–1300 m depth) and at one of these, Hook Ridge, hydrothermal activity and chemosynthetic communities have been observed, making this the most southerly hydrothermal vent site currently known^10,13,15,28^. The sites studied here are at roughly the same depth to eliminate depth as factor from our analysis (Table 2) Hook Ridge 1 was less hydrothermally active than Hook Ridge 2 (hydrothermal advection 9 and 34 cm yr^−1^ respectively) but had greater porewater sulphide^10^. At Hook Ridge 1 there were populations of an endosymbiont-bearing siboglinid tubeworm *Sclerolinum contortum* ^13,17,40^. Bacterial mats were widespread at both sites but patchy^10^, indicating that the flux of sulphide (and other chemosynthetic substrates) to the sediment-water interface was probably similarly patchy. The off-vent site, and others around the Bransfield Strait, received inputs of methane that were putatively thermogenic in origin^16^, suggesting a potential role of sub-surface hydrothermal activity and the δ^13^C signature of this methane corresponded closely to the isotopic signatures of the endosymbiont-bearing species *Siboglinum* spp.^13,16,17^. This may suggest that hydrothermal activity can indirectly support chemosynthetic activity over a much wider area than previously considered but would require additional data (e.g. endosymbiont composition) to more robustly assess.

**Table 2:** Description of sites^10,11,13,17,33,40^. Levels of hydrothermal activity are given here as relative terms.

Site	Depth (m)	Hydrothermally active?	Approximate Temp. (°C)	Chemosynthetic macrofauna?	Chemosynthetic substrates
Off-Vent (BOV)	1150	No	−1	*Siboglinum* sp.	CH_4_ [up to 7 μmol l^−1^]
Hook Ridge 1 (HR1)	1174	Yes, low activity 9 cm yr^−1^ fluid advection^10^	24	*Sclerolinum contortum*	H_2_S [up to 6 μmol l^−1^], CH_4_ [up to 10 μmol l^−1^]
Hook Ridge 2 (HR2)	1054	Yes, high activity 34 cm yr^−1^ fluid advection	48	No	H_2_S [up to 160 μmol l^−1^], CH_4_ [up to 26 μmol l^−1^]

#### Megafauna

Macrofaunal, microbial and geochemical data specific to these sites are published in separate papers^10,11,13,17^. As the methodology for collecting megafaunal assemblage data has not been published yet, it is given here. Seafloor images were collected during Seabed High-Resolution Imaging Platform (SHRIMP) tows conducted during RRS *James Cook* cruise 55 (JC55)^51^. These images were extracted from video footage at a rate of 1 per 10 seconds and analysed for megafaunal abundance in ImageJ^52^. A subset of suitable non-overlapping images were selected^53^ from two areas of Hook Ridge, close to megacore deployment positions^13,17^, totalling a minimum of 150 m^2^ of seafloor imagery per site. There was no SHRIMP tow for the control site on JC55, so density was estimated from measurements at another non-vent site (the Three Sisters), which had comparable macrofaunal composition and density, and sediment organic carbon content^13^. Each image was scaled using the SHRIMP parallel lasers (10 cm apart) and the abundance and areal extent of the fauna; the vast majority being ophiuroids (cf. *Ophionotus* sp. & *Opioperla*s s﻿p.) and holothurians (cf. *Peniagone* sp.), were measured to give density and area per m^2^. Biomass was calculated using fixed relationships between area and dry weight for each group, measured from Agassiz trawl samples collected from Hook Ridge^51^.

### Sediment Community Oxygen Consumption

Sediment community oxygen consumption was measured from overlying water in recovered megacores on board RRS *James Cook* cruise 55. Measurements were made from sealed cores, incubated for 60 hours at bottom temperature with top water continually being stirred, measured as the change in dissolved oxygen per unit time and area, using a Loligo Systems potentiometric sensor.

#### Model Selection

The linear inverse modelling framework was selected because of the considerable uncertainty that needed to be addressed within the analysis. A LIM approach was able to incorporate the wide range of data sources available and directly include the residual uncertainty. The models were designed to address questions concerning the extent of chemosynthetic activity in the Bransfield Strait and the degree to which primary productivity may have been providing trophic subsidies to the local benthos. By calculating the exchange rates between various compartments, a LIM can provide this information, within the a-priori solution space that is outlined by the uncertainty in the parameters supplied.

#### Model Structure and Data Availability

The food web model underlying the LIM was set up as a number of compartments (e.g. detritus, macrofaunal endosymbiont-bearing fauna, megafaunal deposit feeders; Table 3) and each was connected to other compartments by a number of flows^29,31,54^ that reflected their feeding mode(s) and production of detritus or DOC. Compartment biomass was given as the sum of the average carbon masses per taxa, multiplied by density per square metre, using data for dry mass and percentage carbon of faunal tissue collected during preparation and analysis of stable isotopic samples^17^. Carbon stable isotopic data were averaged for each compartment^17^ and used to constrain possible diet composition. By imposing a number of constraints using available data (e.g. biomass or respiration; Table 4), the magnitude of every flow is constrained to a finite and ecologically feasible range of possible values. In addition to the explicit constraints, data provided for each component for biomass and respiration provided implicit constraints upon the magnitude of flows in and out of a given stock (Table 3). External compartments were in some instances given a range of possible values (e.g. POC flux) but others were constrained only by processes occurring between internal compartments (e.g. external predation). The resultant models were used to create large numbers of independently valid solutions, from which the mean and error distribution of each of the unknown flows were calculated^42^.

**Table 3:** Compartments used in the models. Stocks measured in mmol C m^−2^ (e.g. macrofaunal biomass) and rates measured in mmol C m^−2^ d^−1^ (e.g. respiration rates). For compartments where stocks/rates were not defined in the model set up there were no available data (e.g. DIC). Therefore, flows in and out of these compartments were only indirectly determined by constraints upon other compartments and more general production relationships (e.g. biomass-dependent respiration being the main source of DIC). Detritus is termed as any non-living organic material including faecal material, dead bacterial or metazoan tissue and extra polymeric substances like mucus. No data were available to discriminate lability of detrital OM.

Compartment	Code	Depth (bsf)	References (Rates & Stocks)
Internal (exchanges between these compartments determined by permissible flows)			
Detritus	Det	0‒10 cm	10,13,17
Dissolved Organic Carbon	DOC	0‒10 cm	Stock size not defined a-priori, assumed non-limiting
Heterotrophic Bacteria	Bac	0‒1 cm	17
Chemosynthetic Bacteria	ChBac	0‒1 cm	17
Macrofauna with Endosymbionts	MacES	0‒10 cm	13,17
Macrofaunal Deposit Feeders	MacDF	0‒10 cm	13,17
Macrofaunal Suspension Feeders	MacSF	0‒10 cm	13,17
Macrofaunal Predators/Scavengers	MacPS	0‒10 cm	13,17
Megafaunal Deposit Feeders	MegDF	0 cm	This study
Megafaunal Suspension Feeders	MegSF	0 cm	This study
External (inputs to and losses from internal compartments)			
Buried Detritus	Det_s	>10 cm	62
Megafaunal Predation	Predation	Above sediment surface	Loss only, rate not directly constrained a-priori
Biomass specific Respiration + Maintenance Respiration	Respiration	0‒10 cm	13,63
Dissolved Inorganic Carbon	DIC	Not relevant	Stock size not defined a-priori, assumed non-limiting
Dissolved Organic Carbon in the water column	DOC_w	Above sediment surface	Loss only, rate proportional to total respiration^64^
Particulate flux of detritus from the water column	Det_w	Above sediment surface	17,34–37

**Table 4:** Constraints implemented for each model. Parameters contained within [] represent minimum and maximum values that encompass uncertainty in the data. Parameters used marked by ^a, b^ or ^c^ were used specifically for the off-vent site and the low and high activity vent sites respectively. Faunal respiration was calculated separately for each functional group.

Constraint	Unit	Value	Ref.
Deposition of Organic Carbon	mmol C m^−2^ d^−1^	[0.70, 27.17]	34–37
Total Sediment Community Oxygen Consumption	mmol C m^−2^ d^−1^	[0.81, 2.86]^a^ [1.62, 2.86]^b, c^	This study
Relative DOC efflux	‒	[0, 0.1]	64
Q10	‒	2	41,65,66
Temperature limits = Q10^((Temp°C-20)/10)^	‒	0.20^a^[−1 °C], 1.30^b^ [24 °C], 7.00^c^ [48 °C]	29,33
Burial efficiency of Organic C	‒	[0.01, 0.03]	62
Bacterial Growth Efficiency	‒	[0.05, 0.45]	67
Viral lysis of Bacteria (fraction of bacterial production)	‒	[0.30, 0.80]	54,68,69
Efficiency of Chemosynthetic OM fixation	‒	[0.10, 0.50]	70–72
Macrofaunal Growth	‒	[Tlim*0.01, Tlim*0.05]	54
Macrofaunal Net Growth Efficiency	‒	[0.30, 0.70]	54
Macrofaunal Assimilation Efficiency	‒	[0.20, 0.75]	54
Macrofaunal Faecal Production	‒	[0.25, 0.80]	54
Macrofaunal Maintenance Respiration	mmol C m^−2^ d^−1^	Tlim*0.01*Biomass	54
Macrofaunal Respiration	mmol C m^−2^ d^−1^	[0.5, 1.5]* Biomass* Biomass-specific respiration*Tlim	13,63
Megafaunal Growth	‒	[Tlim*0.0027, Tlim*0.014]	54
Megafaunal Net Growth Efficiency	‒	[0.50, 0.70]	54
Megafaunal Assimilation Efficiency	‒	[0.20, 0.75]	54
MegafaunalFaecal Production	‒	[0.25, 0.80]	54
Megafaunal Maintenance Respiration	mmol C m^−2^ d^−1^	Tlim*0.001*Biomass	54
Megafaunal Respiration	mmol C m^−2^ d^−1^	[0.5, 1.5]* Biomass* Biomass-specific respiration*Tlim	13,63

Where site-specific data were not available, we used constraints (e.g. bacterial growth efficiency) that encompassed a typical range from a wider variety of studies to constrain the flows in the network (Table 4). This approach is consistent with previous implementations of this modelling framework in the deep-sea, where such data are rarely available for specific environments^29–31^. Bacterial biomass was estimated for the more active vent site (HR2) from isotopically labelled samples, since comparable natural phospholipid fatty acid data were not available^17^. At other sites, natural bacterial biomass was 45–60% of that measured in pulse-chase experiments and we used an estimate, based on bacterial biomass from labelled samples collected at HR2^17^.

### LIM Implementation

Each of these models were implemented into the LIM framework in the R statistical environment^55^ in the package LIM^42,56^ using the data outlined above and a series of equality (a = b) and inequality (a > b) constraints. The model structure (constraints and flows) was the same for each site so the differences observed resulted from differences in biomass, respiration and stable isotopic signatures.

#### Model Solutions

To determine the number of model iterations required to achieve a representative series of mean flows for each site, we compared the means of several flows between data from a series of model solutions with different numbers of iterations. Each model was sampled for 300, 3 000, 30 000 and 200 000 iterations to compare change in mean and standard deviation with increasing replication for a subset of flows of varying magnitude. Each set of solutions was begun from the same set of approximated flow means for both the preliminary tests and the final solutions. The minimum number of solutions required was determined at the point where the mean and standard deviations had converged to within 2% of the final value given by the most sampled dataset. Most flows were within this range within 1000 iterations, but some flows that had higher standard deviation required more than 30 000 iterations to converge. The minimum number of iterations required to reach convergence (±2%), for all of the 5 flows sampled, was approximately 46 000, 37 000 and 13 000 for the off-vent site and the low and high hydrothermal activity sites respectively. In the final set of model outcomes presented here, we used approximately double the greatest number of iterations required (detritus production by macrofaunal deposit feeders at BOV). Thus the final set of solutions consisted of 100 000 iterations for each of the three models from which means and standard deviations for each flow were calculated^42^. The reason for using this likelihood approach^29,42^ is that the fitted LIM still had an infinitely large number of possible solutions, each one corresponding to an independently valid solution (individual flow values are not necessarily exchangeable between solutions).

We calculated network summary statistics for each of the model solutions using the R package ‘NetIndices’^30,57–60^. These indices were calculated to compare network structure (i.e. the degree of compartmentalisation, number of links or average mutual information) and the proportion of flows that were recycled (Finn cycling index)at each site. Average Mutual Information measures the average constraint upon a each flow and tends towards lower values in more mature ecosystems^60^.

Since this approach results in a large solution set (n = 10^5^), conventional comparison tests were highly sensitive to very small differences between variables. Several results had very low significance values but with negligible effect sizes (Cohen’s D), such as differences in POC deposition (pairwise Wilcoxon test between HR1 and HR2: p < 2 × 10^−16^, Cohen’s D estimate = 0.03)^61^. Data were also not normally distributed and had unequal variance between models. Therefore, we compared variables by calculating the fraction of solutions from a particular solution set that were larger than either of the other two. We define differences where >95% of the estimates from one solution were greater than from another as being significant and >98% as being highly significant^30^.

#### Model solutions and error distribution

The quality of the final model solution sets was evaluated using the coefficient of variation (CoV, standard deviation divided by mean) for each flow. CoV provides a simple indication of residual uncertainty within the solution space and flows with a CoV of 1 or more (i.e. standard deviation equals or exceeds the mean) can be considered as still having considerable uncertainty. Mean CoV across all flows for BOV (31 flows), HR1 (38 flows) and HR2 (37 flows) was 0.41, 0.44 and 0.29 respectively, indicating that error distribution each of the models was generally quite low. Maximum CoV values were 0.70, 0.90 and 0.95 respectively but 60.5–80.4% of flows had CoV values of <0.5 and 10.5–51.4% of flows have CoV values of <0.2, demonstrating that the majority of values had quite small errors, relative to their means.

#### Model limitations

Despite a large dataset, data for some compartments used in some other LIM studies e.g.^29^ were not available. Meiofaunal data were lacking entirely and as such were excluded from the model. Transfers between bacteria and metazoa were mediated by meiofaunal activity to some degree but we could not explicitly investigate this activity. Detailed data on the composition of detritus were also not available and thus detritus was considered a single compartment, with no consideration given to OM lability^29^. Additionally, stable isotopic data were used to inform dietary proportions but were averaged for whole compartments so may have contributed increased uncertainty to some of the results (such as bacteria to macrofaunal predator/scavengers at the off-vent site).

## Electronic supplementary material

Supplementary Dataset 1
