# Supplementary material for: Hydrothermal activity, functional diversity and chemoautotrophy are major drivers of seafloor carbon cycling
Source: Sci Rep. 2017 Sep 20;7:12025. doi: 10.1038/s41598-017-12291-w (PMC5607325; doi:10.1038/s41598-017-12291-w)
Supplement: Supplementary file 1 — Supplementary Dataset 1 [file 41598_2017_12291_MOESM1_ESM.doc]

# Supplementary information for ‘Hydrothermal activity, functional diversity and chemoautotrophy are major drivers of seafloor carbon cycling.’

James B. Bell^1, 2^, Clare Woulds^1^*& Dick van Oevelen^3^

Data : LIM model for Bransfield Strait Sedimented Vents, sampled Jan 2011

Site : Bransfield Control

Units :

Fluxes : mmol C m-2 d-1

Stocks : mmol C m-2

Depth : upper 10 cm

########

!---------------------------------------------------------------------!

! !

! PARAMETER & VARIABLES !

! !

!---------------------------------------------------------------------!

## PARAMETERS

!Site-specific data

! Flows

minPOCdeposition = 0.70 {mmol C m-2 d-1}

maxPOCdeposition = 27.17 {mmol C m-2 d-1}

minTotResp = 0.81 {mmol C m-2 d-1}

maxTotResp = 2.86 {mmol C m-2 d-1}

Tlim = 0.20 {-}

! Stocks

Detritus = 114.47 {mmol C m-2}

HeterotrophicBac = 11.21 {mmol C m-2}

FreeChemoautotrophicBac = 0.00 {mmol C m-2}

MacroEndosymbiotic = 0.56 {mmol C m-2}

MacroDepositFeeder = 73.08 {mmol C m-2}

MacroSuspensionFeeder = 2.04 {mmol C m-2}

MacroPredatorScavenger = 14.48 {mmol C m-2}

MegaDepositFeeder = 21.04 {mmol C m-2}

MegaSuspensionFeeder = 0.00 {mmol C m-2}

! Bacterial Respiration

ChBacResp = 0.00 {mmol C m-2 d-1}

! Macro Respiration

TotRespMacDF = 0.765*Tlim {mmol C m-2 d-1} !total respiration

TotRespMacSF = 0.019*Tlim {mmol C m-2 d-1}

TotRespMacPS = 0.128*Tlim {mmol C m-2 d-1}

TotRespMacES = 0.009*Tlim {mmol C m-2 d-1}

! Mega Respiration

TotRespMegDF = 0.044*Tlim {mmol C m-2 d-1}

TotRespMegSF = 0.000*Tlim {mmol C m-2 d-1}

! Methane Flux

minSulphideFlux = 0.00 {mmol C m-2 d-1}

maxSulphideFlux = 1.00 {mmol C m-2 d-1}

! Stable Isotope Data

13C_Det_w = -27.0 {delta 13C}

13C_Det = -25.9 {delta 13C}

13C_Bac = -26.8 {delta 13C}

13C_ChBac = 0.0 {delta 13C}

13C_MacES = -45.3 {delta 13C}

13C_MacDF = -25.9 {delta 13C}

13C_MacSF = 0.0 {delta 13C}

13C_MacPS = -26.9 {delta 13C}

13C_MegDF = -26.1 {delta 13C}

13C_MegSF = -27.0 {delta 13C}

! Generic Data

! Detritus

minBur = 0.01 {-}

maxBur = 0.03 {-}

maxRelDOCefflux = 0.10 {-}

! Bacteria

minBGE = 0.05 {-}

maxBGE = 0.45 {-}

minLysBP = 0.30 {-}

maxLysBP = 0.80 {-}

minChemoEff = 0.10 {-}

maxChemoEff = 0.50 {-}

! Macrofauna

minGrowthMac = Tlim*0.01 {d-1}

maxGrowthMac = Tlim*0.05 {d-1}

minMacAss = 0.20 {-} !assimilation efficiency

maxMacAss = 0.75 {-}

minMacNGE = 0.30 {-} !net growth efficiency

maxMacNGE = 0.70 {-}

minMacExcrete = 0.25 {-}

maxMacExcrete = 0.80 {-}

MacRespUpper = 1.50 {-}

MacRespLower = 0.50 {-}

MaintRespMacDF = Tlim*0.001*MacroDepositFeeder {mmol C m-2 d-1}

MaintRespMacSF = Tlim*0.001*MacroSuspensionFeeder {mmol C m-2 d-1}

MaintRespMacPS = Tlim*0.001*MacroPredatorScavenger {mmol C m-2 d-1}

MaintRespMacES = Tlim*0.001*MacroEndosymbiotic {mmol C m-2 d-1}

! Megafauna

minGrowthMeg = Tlim*0.0027 {d-1}

maxGrowthMeg = Tlim*0.014 {d-1}

minMegAss = 0.20 {-} !assimilation efficiency

maxMegAss = 0.75 {-}

minMegExcrete = 0.25 {-}

maxMegExcrete = 0.80 {-}

minMegNGE = 0.50 {-} !net growth efficiency

maxMegNGE = 0.70 {-}

MegRespUpper = 1.50 {-}

MegRespLower = 0.50 {-}

MaintRespMegDF = Tlim*0.00001*MegaDepositFeeder {mmol C m-2 d-1}

MaintRespMegSF = Tlim*0.00001*MegaSuspensionFeeder {mmol C m-2 d-1}

## END PARAMETERS

### VARIABLES

!-- Detritus

OMInput = Det_w -> Det + Det_w -> MacSF + Det_w -> MegSF + ChBacProd + MacESProd

POCdeposition = Det_w -> Det

DetDis = Det -> DOC

BurialOC = Det -> Det_s

! DOC

DOCefflux = DOC -> DOC_w

! Bacteria

BacResp = Bac -> Respiration

BacUpt = DOC -> Bac

BacDocProd = Bac -> DOC

BacProd = BacUpt - BacResp

ChBacResp = ChBac -> Respiration

ChBacUpt = flowto(ChBac)

ChBacDocProd = ChBac -> DOC

ChBacProd = ChBacUpt - ChBacResp

! Macrofauna

MacESUpt = flowto(MacES)

MacESProd = MacESUpt - MacESResp

MacESResp = MacES -> Respiration

MacEsDetProd = MacES -> Det

MacESAss = MacESupt - MacESDetProd

MacESPro = MacESAss - MacESResp

MacDFResp = MacDF -> Respiration

MacDFDetProd = MacDF -> Det

MacDFupt = flowto(MacDF)

MacDFAss = MacDFupt - MacDFDetProd

MacDFPro = MacDFAss - MacDFResp

MacDFGrowth = MacDFAss - MacDFresp + MaintRespMacDF

MacSFResp = MacSF -> Respiration

MacSFDetProd = MacSF -> Det

MacSFupt = flowto(MacSF)

MacSFAss = MacSFupt - MacSFDetProd

MacSFPro = MacSFAss - MacSFResp

MacSFGrowth = MacSFAss - MacSFresp + MaintRespMacSF

MacPSResp = MacPS -> Respiration

MacPSDetProd = MacPS -> Det

MacPSupt = flowto(MacPS)

MacPSAss = MacPSupt - MacPSDetProd

MacPSPro = MacPSAss - MacPSResp

! Megafauna

MegDFResp = MegDF -> Respiration

MegDFDetProd = MegDF -> Det

MegDFupt = flowto(MegDF)

MegDFAss = MegDFupt - MegDFDetProd

MegDFPro = MegDFAss - MegDFResp

MegDFGrowth = MegDFAss - MegDFresp + MaintRespMegDF

MegSFResp = MegSF -> Respiration

MegSFDetProd = MegSF -> Det

MegSFupt = flowto(MegSF)

MegSFAss = MegSFupt - MegSFDetProd

MegSFPro = MegSFAss - MegSFResp

MegSFGrowth = MegSFAss - MegSFresp + MaintRespMegSF

! Totals

BacterialResp = BacResp + ChBacResp

MacResp = MacDFResp + MacSFResp + MacPSResp + MacESResp

MegResp = MegDFResp + MegSFResp

Suspensionfeeding = Det_w -> MacSF + Det_w -> MegSF

NetSuspensionfeeding = Suspensionfeeding - MacSFResp - MegSFResp

TotInSituProd = DIC -> ChBac + DIC -> MacES

NetInSituInput = ChBacProd + MacESPro

MacPredation = MacES -> Predation + MacDF -> Predation + MacSF -> Predation + MacPS -> Predation

MegPredation = MegDF -> Predation + MegSF -> Predation

TotPredation = MacPredation + MegPredation

ExtPredation = flowto(Predation)

TotResp = BacterialResp + MacResp + MegResp

TotSinkingOM = POCdeposition + Suspensionfeeding

NetSinkingOMInput = POCdeposition + NetSuspensionfeeding

TotOMinput = POCdeposition + Suspensionfeeding + TotInSituProd

NetTotOMInput = NetInSituInput + NetSinkingOMInput

TotDetProd = flowto(Det)

#### END VARIABLES

!-------------------------------------------------------------------!

! !

! SYSTEM DECLARATION !

! (STOCKS, EXTERNALS, FLOWS) !

! !

!-------------------------------------------------------------------!

#### STOCK

Det = Detritus

DOC

Bac = HeterotrophicBac

ChBac = FreeChemoautotrophicBac

MacES = MacroEndosymbiotic

MacDF = MacroDepositFeeder

MegDF = MegaDepositFeeder

MacPS = MacroPredatorScavenger

MacSF = MacroSuspensionFeeder

MegSF = MegaSuspensionFeeder

##### END STOCK

##### EXTERNALS

Det_s !Burial

Predation !e.g. Fish/ Megafaunal Predators

Respiration

DIC

DOC_w

Det_w

##### END EXTERNALS

##### FLOW

! Detritus production/ Mortality

Det_w -> Det

DOC -> DOC_w

Det -> DOC

Bac -> DOC

ChBac -> DOC

MacDF -> Det

MacES -> Det

MacSF -> Det

MacPS -> Det

MegDF -> Det

MegSF -> Det

! Bacteria

DIC -> ChBac

DOC -> Bac

! Macrofauna

DIC -> MacES

Det -> MacDF

ChBac -> MacDF

Bac -> MacDF

Det_w -> MacSF

Bac -> MacPS

ChBac -> MacPS

MacES -> MacPS

MacDF -> MacPS

MacSF -> MacPS

! Megafauna

Det -> MegDF

ChBac -> MegDF

Bac -> MegDF

Det_w -> MegSF

! Respiration

Bac -> Respiration

ChBac -> Respiration

MacES -> Respiration

MacDF -> Respiration

MacSF -> Respiration

MacPS -> Respiration

MegDF -> Respiration

MegSF -> Respiration

! Predation

MacES -> Predation

MacDF -> Predation

MacSF -> Predation

MacPS -> Predation

MegDF -> Predation

MegSF -> Predation

! Burial

Det -> Det_s

###### END FLOW

!------------------------------------------------------------------!

! !

! DATA DECLARATION !

! (RATES, EQUATIONS, CONSTRAINTS) !

! !

!------------------------------------------------------------------!

####### RATE

! all rates zero

##### END RATE

##### EQUATION

Flow(Det,MacDF)*13C_Det + Flow(ChBac,MacDF)*13C_ChBac + Flow(Bac,MacDF)*13C_Bac - &

Flow(Det,MacDF)*13C_MacDF - Flow(ChBac,MacDF)*13C_MacDF - Flow(Bac,MacDF)*13C_MacDF = 0

Flow(Det,MegDF)*13C_Det + Flow(ChBac,MegDF)*13C_ChBac + Flow(Bac,MegDF)*13C_Bac - &

Flow(Det,MegDF)*13C_MegDF - Flow(ChBac,MegDF)*13C_MegDF - Flow(Bac,MegDF)*13C_MegDF = 0

Flow(Bac,MacPS)*13C_Bac + Flow(MacSF,MacPS)*13C_MacSF + Flow(ChBac,MacPS)*13C_ChBac + &

Flow(MacES,MacPS)*13C_MacES + Flow(MacDF,MacPS)*13C_MacDF - &

Flow(Bac,MacPS)*13C_MacPS - Flow(MacSF,MacPS)*13C_MacPS - Flow(ChBac,MacPS)*13C_MacPS - &

Flow(MacES,MacPS)*13C_MacPS - Flow(MacDF,MacPS)*13C_MacPS = 0

###### END EQUATION

##### CONSTRAINT

! POC

POCdeposition = [minPOCdeposition,maxPOCdeposition]

! Totals

TotResp = [minTotResp,maxTotResp]

! Bacteria

ChBacUpt = [minChemoEff, maxChemoEff]*[minSulphideFlux, maxSulphideFlux]

ChBacProd = [minBGE,MaxBGE]*ChBacUpt

ChBacDocProd = [minLysBP,maxLysBP]*ChBacProd

BacProd = [minBGE,MaxBGE]*BacUpt

BacDocProd = [minLysBP,maxLysBP]*BacProd

! Macrofauna ES

MacESUpt = [minChemoEff, maxChemoEff]*[minSulphideFlux, maxSulphideFlux]

MacESAss - MacESresp + MaintRespMacES = [minMacNGE, maxMacNGE]*MacESAss

MacESAss - MacESResp + MaintRespMacES = [minGrowthMac, maxGrowthMac]*MacES

! Macrofauna DF

MacDFresp = [MacRespLower, MacRespUpper]*TotRespMacDF

MacDFAss = [minMacAss, maxMacAss]*MacDFUpt

MacDFDetProd = [minMacExcrete, maxMacExcrete]*MacDFUpt

MacDFAss - MacDFresp + MaintRespMacDF = [minMacNGE, maxMacNGE]*MacDFAss

MacDFAss - MacDFResp + MaintRespMacDF = [minGrowthMac, maxGrowthMac]*MacDF

! Macrofauna SF

MacSFresp = [MacRespLower, MacRespUpper]*TotRespMacSF

MacSFAss = [minMacAss, maxMacAss]*MacSFUpt

MacSFDetProd = [minMacExcrete, maxMacExcrete]*MacSFUpt

MacSFAss - MacSFresp + MaintRespMacSF = [minMacNGE, maxMacNGE]*MacSFAss

MacSFAss - MacSFResp + MaintRespMacSF = [minGrowthMac, maxGrowthMac]*MacSF

! Macrofauna PS

MacPSresp = [MacRespLower, MacRespUpper]*TotRespMacPS

MacPSAss = [minMacAss, maxMacAss]*MacPSUpt

MacPSDetProd = [minMacExcrete, maxMacExcrete]*MacPSUpt

MacPSAss - MacPSresp + MaintRespMacPS = [minMacNGE, maxMacNGE]*MacPSAss

MacPSAss - MacPSResp + MaintRespMacPS = [minGrowthMac, maxGrowthMac]*MacPS

! Megafauna DF

MegDFresp = [MegRespLower, MegRespUpper]*TotRespMegDF

MegDFAss = [minMegAss, maxMegAss]*MegDFUpt

MegDFDetProd = [minMegExcrete, maxMegExcrete]*MegDFUpt

MegDFAss - MegDFresp + MaintRespMegDF = [minMegNGE, maxMegNGE]*MegDFAss

MegDFAss - MegDFResp + MaintRespMegDF = [minGrowthMeg, maxGrowthMeg]*MegDF

! Megafauna SF

MegSFresp = [MegRespLower, MegRespUpper]*TotRespMegSF

MegSFAss = [minMegAss, maxMegAss]*MegSFUpt

MegSFDetProd = [minMegExcrete, maxMegExcrete]*MegSFUpt

MegSFAss - MegSFresp + MaintRespMegSF = [minMegNGE, maxMegNGE]*MegSFAss

MegSFAss - MegSFResp + MaintRespMegSF = [minGrowthMeg, maxGrowthMeg]*MegSF

!-- Efflux of DOC from the sediment

DOCefflux < maxRelDOCefflux*TotResp !Burdige GCA 63:1507-1515

! Burial

BurialOC = [minBur, maxBur]*TotOMinput

##### END CONSTRAINT

Data : LIM model for Bransfield Strait Sedimented Vents, sampled Jan 2011

Site : Hook Ridge 1

Units :

Fluxes : mmol C m-2 d-1

Stocks : mmol C m-2

Depth : upper 10 cm

########

!---------------------------------------------------------------------!

! !

! PARAMETER & VARIABLES !

! !

!---------------------------------------------------------------------!

## PARAMETERS

!Site specific Data

! Flows

minPOCdeposition = 0.70 {mmol C m-2 d-1}

maxPOCdeposition = 27.17 {mmol C m-2 d-1}

minTotResp = 1.62 {mmol C m-2 d-1}

maxTotResp = 2.86 {mmol C m-2 d-1}

minChemoFix = 2.80 {mmol C m-2 d-1}

maxChemoFix = 16.80 {mmol C m-2 d-1}

Tlim = 1.30 {-}

! Stocks

Detritus = 114.47 {mmol C m-2}

HeterotrophicBac = 13.82 {mmol C m-2}

FreeChemoautotrophicBac = 30.73 {mmol C m-2}

MacroEndosymbiotic = 4.46 {mmol C m-2}

MacroDepositFeeder = 25.51 {mmol C m-2}

MacroSuspensionFeeder = 5.98 {mmol C m-2}

MacroPredatorScavenger = 4.18 {mmol C m-2}

MegaDepositFeeder = 27.45 {mmol C m-2}

MegaSuspensionFeeder = 0.00 {mmol C m-2}

! Macro Respiration

TotRespMacDF = 0.33*Tlim {mmol C m-2 d-1} !total respiration

TotRespMacSF = 0.06*Tlim {mmol C m-2 d-1}

TotRespMacPS = 0.05*Tlim {mmol C m-2 d-1}

TotRespMacES = 0.11*Tlim {mmol C m-2 d-1}

! Mega Respiration

TotRespMegDF = 0.064*Tlim {mmol C m-2 d-1}

TotRespMegSF = 0.000*Tlim {mmol C m-2 d-1}

! Stable Isotope Data

13C_Det_w = -27.0 {delta 13C}

13C_Det = -25.6 {delta 13C}

13C_Bac = -26.6 {delta 13C} !weighted average less PLFA offset

13C_ChBac = -20.4 {delta 13C}

13C_MacES = -20.7 {delta 13C}

13C_MacDF = -25.2 {delta 13C}

13C_MacSF = -26.8 {delta 13C}

13C_MacPS = -23.9 {delta 13C}

13C_MegDF = -26.1 {delta 13C}

13C_MegSF = 0.0 {delta 13C}

! Sulphide Flux

minSulphideFlux = 0.08 {mmol C m-2 d-1}

maxSulphideFlux = 7.45 {mmol C m-2 d-1}

! Generic Data

! Detritus

minBur = 0.01 {-}

maxBur = 0.03 {-}

maxRelDOCefflux = 0.10 {-}

! Bacteria

minBGE = 0.05 {-}

maxBGE = 0.45 {-}

minLysBP = 0.30 {-}

maxLysBP = 0.80 {-}

minChemoEff = 0.10 {-}

maxChemoEff = 0.50 {-}

! Macrofauna

minGrowthMac = Tlim*0.01 {d-1}

maxGrowthMac = Tlim*0.05 {d-1}

minMacAss = 0.20 {-} !assimilation efficiency

maxMacAss = 0.75 {-}

minMacNGE = 0.3 {-} !net growth efficiency

maxMacNGE = 0.7 {-}

minMacExcrete = 0.25 {-}

maxMacExcrete = 0.80 {-}

MacRespUpper = 1.5 {-}

MacRespLower = 0.5 {-}

MaintRespMacDF = Tlim*0.001*MacroDepositFeeder {mmol C m-2 d-1}

MaintRespMacSF = Tlim*0.001*MacroSuspensionFeeder {mmol C m-2 d-1}

MaintRespMacPS = Tlim*0.001*MacroPredatorScavenger {mmol C m-2 d-1}

MaintRespMacES = Tlim*0.001*MacroEndosymbiotic {mmol C m-2 d-1}

! Megafauna

minGrowthMeg = Tlim*0.0027 {d-1}

maxGrowthMeg = Tlim*0.014 {d-1}

minMegAss = 0.20 {-} !assimilation efficiency

maxMegAss = 0.75 {-}

minMegExcrete = 0.25 {-}

maxMegExcrete = 0.80 {-}

minMegNGE = 0.5 {-} !net growth efficiency

maxMegNGE = 0.7 {-}

MegRespUpper = 1.5 {-}

MegRespLower = 0.5 {-}

MaintRespMegDF = Tlim*0.00001*MegaDepositFeeder {mmol C m-2 d-1}

MaintRespMegSF = Tlim*0.00001*MegaSuspensionFeeder {mmol C m-2 d-1}

## END PARAMETERS

### VARIABLES

!-- Detritus

OMInput = Det_w -> Det + Det_w -> MacSF + Det_w -> MegSF + ChBacProd + MacESProd

POCdeposition = Det_w -> Det

DetDis = Det -> DOC

BurialOC = Det -> Det_s

! DOC

DOCefflux = DOC -> DOC_w

! Bacteria

BacResp = Bac -> Respiration

BacUpt = DOC -> Bac

BacDocProd = Bac -> DOC

BacProd = BacUpt - BacResp

ChBacResp = ChBac -> Respiration

ChBacUpt = flowto(ChBac)

ChBacDocProd = ChBac -> DOC

ChBacProd = ChBacUpt - ChBacResp

! Macrofauna

MacESUpt = flowto(MacES)

MacESProd = MacESUpt - MacESResp

MacESResp = MacES -> Respiration

MacEsDetProd = MacES -> Det

MacESAss = MacESupt - MacESDetProd

MacESPro = MacESAss - MacESResp

MacDFResp = MacDF -> Respiration

MacDFDetProd = MacDF -> Det

MacDFupt = flowto(MacDF)

MacDFAss = MacDFupt - MacDFDetProd

MacDFPro = MacDFAss - MacDFResp

MacDFGrowth = MacDFAss - MacDFresp + MaintRespMacDF

MacSFResp = MacSF -> Respiration

MacSFDetProd = MacSF -> Det

MacSFupt = flowto(MacSF)

MacSFAss = MacSFupt - MacSFDetProd

MacSFPro = MacSFAss - MacSFResp

MacSFGrowth = MacSFAss - MacSFresp + MaintRespMacSF

MacPSResp = MacPS -> Respiration

MacPSDetProd = MacPS -> Det

MacPSupt = flowto(MacPS)

MacPSAss = MacPSupt - MacPSDetProd

MacPSPro = MacPSAss - MacPSResp

! Megafauna

MegDFResp = MegDF -> Respiration

MegDFDetProd = MegDF -> Det

MegDFupt = flowto(MegDF)

MegDFAss = MegDFupt - MegDFDetProd

MegDFPro = MegDFAss - MegDFResp

MegDFGrowth = MegDFAss - MegDFresp + MaintRespMegDF

MegSFResp = MegSF -> Respiration

MegSFDetProd = MegSF -> Det

MegSFupt = flowto(MegSF)

MegSFAss = MegSFupt - MegSFDetProd

MegSFPro = MegSFAss - MegSFResp

MegSFGrowth = MegSFAss - MegSFresp + MaintRespMegSF

! Totals

BacterialResp = BacResp + ChBacResp

MacResp = MacDFResp + MacSFResp + MacPSResp + MacESResp

MegResp = MegDFResp + MegSFResp

Suspensionfeeding = Det_w -> MacSF + Det_w -> MegSF

NetSuspensionfeeding = Suspensionfeeding - MacSFResp - MegSFResp

TotInSituProd = DIC -> ChBac + DIC -> MacES

NetInSituInput = ChBacProd + MacESPro

MacPredation = MacES -> Predation + MacDF -> Predation + MacSF -> Predation + MacPS -> Predation

MegPredation = MegDF -> Predation + MegSF -> Predation

TotPredation = MacPredation + MegPredation

ExtPredation = flowto(Predation)

TotResp = BacterialResp + MacResp + MegResp

TotSinkingOM = POCdeposition + Suspensionfeeding

NetSinkingOMInput = POCdeposition + NetSuspensionfeeding

TotOMinput = POCdeposition + Suspensionfeeding + TotInSituProd

NetTotOMInput = NetInSituInput + NetSinkingOMInput

TotDetProd = flowto(Det)

#### END VARIABLES

!-------------------------------------------------------------------!

! !

! SYSTEM DECLARATION !

! (STOCKS, EXTERNALS, FLOWS) !

! !

!-------------------------------------------------------------------!

#### STOCK

Det = Detritus

DOC

Bac = HeterotrophicBac

ChBac = FreeChemoautotrophicBac

MacES = MacroEndosymbiotic

MacDF = MacroDepositFeeder

MegDF = MegaDepositFeeder

MacPS = MacroPredatorScavenger

MacSF = MacroSuspensionFeeder

MegSF = MegaSuspensionFeeder

##### END STOCK

##### EXTERNALS

Det_s !Burial

Predation !e.g. Fish/ Megafaunal Predators

Respiration

DIC

DOC_w

Det_w

##### END EXTERNALS

##### FLOW

! Detritus production/ Mortality

Det_w -> Det

DOC -> DOC_w

Det -> DOC

Bac -> DOC

ChBac -> DOC

MacDF -> Det

MacES -> Det

MacSF -> Det

MacPS -> Det

MegDF -> Det

MegSF -> Det

! Bacteria

DIC -> ChBac

DOC -> Bac

! Macrofauna

DIC -> MacES

Det -> MacDF

ChBac -> MacDF

Bac -> MacDF

Det_w -> MacSF

Bac -> MacPS

ChBac -> MacPS

MacES -> MacPS

MacDF -> MacPS

MacSF -> MacPS

! Megafauna

Det -> MegDF

ChBac -> MegDF

Bac -> MegDF

Det_w -> MegSF

! Respiration

Bac -> Respiration

ChBac -> Respiration

MacES -> Respiration

MacDF -> Respiration

MacSF -> Respiration

MacPS -> Respiration

MegDF -> Respiration

MegSF -> Respiration

! Predation

MacES -> Predation

MacDF -> Predation

MacSF -> Predation

MacPS -> Predation

MegDF -> Predation

MegSF -> Predation

! Burial

Det -> Det_s

###### END FLOW

!------------------------------------------------------------------!

! !

! DATA DECLARATION !

! (RATES, EQUATIONS, CONSTRAINTS) !

! !

!------------------------------------------------------------------!

####### RATE

! all rates zero

##### END RATE

##### EQUATION

Flow(Det,MacDF)*13C_Det + Flow(ChBac,MacDF)*13C_ChBac + Flow(Bac,MacDF)*13C_Bac - &

Flow(Det,MacDF)*13C_MacDF - Flow(ChBac,MacDF)*13C_MacDF - Flow(Bac,MacDF)*13C_MacDF = 0

Flow(Det,MegDF)*13C_Det + Flow(ChBac,MegDF)*13C_ChBac + Flow(Bac,MegDF)*13C_Bac - &

Flow(Det,MegDF)*13C_MegDF - Flow(ChBac,MegDF)*13C_MegDF - Flow(Bac,MegDF)*13C_MegDF = 0

Flow(Bac,MacPS)*13C_Bac + Flow(MacSF,MacPS)*13C_MacSF + Flow(ChBac,MacPS)*13C_ChBac + &

Flow(MacES,MacPS)*13C_MacES + Flow(MacDF,MacPS)*13C_MacDF - &

Flow(Bac,MacPS)*13C_MacPS - Flow(MacSF,MacPS)*13C_MacPS - Flow(ChBac,MacPS)*13C_MacPS - &

Flow(MacES,MacPS)*13C_MacPS - Flow(MacDF,MacPS)*13C_MacPS = 0

###### END EQUATION

##### CONSTRAINT

! POC

POCdeposition = [minPOCdeposition,maxPOCdeposition]

! Totals

TotResp = [minTotResp,maxTotResp]

! Bacteria

ChBacUpt = [minChemoEff, maxChemoEff]*[minSulphideFlux, maxSulphideFlux]

ChBacProd = [minBGE,MaxBGE]*ChBacUpt

ChBacDocProd = [minLysBP,maxLysBP]*ChBacProd

BacProd = [minBGE,MaxBGE]*BacUpt

BacDocProd = [minLysBP,maxLysBP]*BacProd

! Macrofauna ES

MacESUpt = [minChemoEff, maxChemoEff]*[minSulphideFlux, maxSulphideFlux]

MacESAss - MacESresp + MaintRespMacES = [minMacNGE, maxMacNGE]*MacESAss

MacESAss - MacESResp + MaintRespMacES = [minGrowthMac, maxGrowthMac]*MacES

! Macrofauna DF

MacDFresp = [MacRespLower, MacRespUpper]*TotRespMacDF

MacDFAss = [minMacAss, maxMacAss]*MacDFUpt

MacDFDetProd = [minMacExcrete, maxMacExcrete]*MacDFUpt

MacDFAss - MacDFresp + MaintRespMacDF = [minMacNGE, maxMacNGE]*MacDFAss

MacDFAss - MacDFResp + MaintRespMacDF = [minGrowthMac, maxGrowthMac]*MacDF

! Macrofauna SF

MacSFresp = [MacRespLower, MacRespUpper]*TotRespMacSF

MacSFAss = [minMacAss, maxMacAss]*MacSFUpt

MacSFDetProd = [minMacExcrete, maxMacExcrete]*MacSFUpt

MacSFAss - MacSFresp + MaintRespMacSF = [minMacNGE, maxMacNGE]*MacSFAss

MacSFAss - MacSFResp + MaintRespMacSF = [minGrowthMac, maxGrowthMac]*MacSF

! Macrofauna PS

MacPSresp = [MacRespLower, MacRespUpper]*TotRespMacPS

MacPSAss = [minMacAss, maxMacAss]*MacPSUpt

MacPSDetProd = [minMacExcrete, maxMacExcrete]*MacPSUpt

MacPSAss - MacPSresp + MaintRespMacPS = [minMacNGE, maxMacNGE]*MacPSAss

MacPSAss - MacPSResp + MaintRespMacPS = [minGrowthMac, maxGrowthMac]*MacPS

! Megafauna DF

MegDFresp = [MegRespLower, MegRespUpper]*TotRespMegDF

MegDFAss = [minMegAss, maxMegAss]*MegDFUpt

MegDFDetProd = [minMegExcrete, maxMegExcrete]*MegDFUpt

MegDFAss - MegDFresp + MaintRespMegDF = [minMegNGE, maxMegNGE]*MegDFAss

MegDFAss - MegDFResp + MaintRespMegDF = [minGrowthMeg, maxGrowthMeg]*MegDF

! Megafauna SF

MegSFresp = [MegRespLower, MegRespUpper]*TotRespMegSF

MegSFAss = [minMegAss, maxMegAss]*MegSFUpt

MegSFDetProd = [minMegExcrete, maxMegExcrete]*MegSFUpt

MegSFAss - MegSFresp + MaintRespMegSF = [minMegNGE, maxMegNGE]*MegSFAss

MegSFAss - MegSFResp + MaintRespMegSF = [minGrowthMeg, maxGrowthMeg]*MegSF

!-- Efflux of DOC from the sediment

DOCefflux < maxRelDOCefflux*TotResp !Burdige GCA 63:1507-1515

! Burial

BurialOC = [minBur, maxBur]*TotOMinput

##### END CONSTRAINT

Data : LIM model for Bransfield Strait Sedimented Vents, sampled Jan 2011

Site : Hook Ridge 2

Units :

Fluxes : mmol C m-2 d-1

Stocks : mmol C m-2

Depth : upper 10 cm

########

!---------------------------------------------------------------------!

! !

! PARAMETER & VARIABLES !

! !

!---------------------------------------------------------------------!

## PARAMETERS

! Site-specific data

! Flows

minPOCdeposition = 0.70 {mmol C m-2 d-1}

maxPOCdeposition = 27.17 {mmol C m-2 d-1}

minTotResp = 1.62 {mmol C m-2 d-1}

maxTotResp = 2.86 {mmol C m-2 d-1}

minChemoFix = 2.80 {mmol C m-2 d-1}

maxChemoFix = 16.80 {mmol C m-2 d-1}

Tlim = 7.00 {-}

! Stocks

Detritus = 82.25 {mmol C m-2}

HeterotrophicBac = 13.82 {mmol C m-2} !estimate of ~52% of labelled biomass

FreeChemoautotrophicBac = 6.26 {mmol C m-2}

MacroDepositFeeder = 8.88 {mmol C m-2}

MacroEndosymbiotic = 0.00 {mmol C m-2}

MacroSuspensionFeeder = 13.96 {mmol C m-2}

MacroPredatorScavenger = 0.62 {mmol C m-2}

MegaDepositFeeder = 24.30 {mmol C m-2}

MegaSuspensionFeeder = 0.04 {mmol C m-2}

! Macro Respiration

TotRespMacDF = 0.10*Tlim {mmol C m-2 d-1} !total respiration

TotRespMacSF = 0.12*Tlim {mmol C m-2 d-1}

TotRespMacPS = 0.04*Tlim {mmol C m-2 d-1}

TotRespMacES = 0*Tlim {mmol C m-2 d-1}

! Mega Respiration

TotRespMegDF = 0.042*Tlim {mmol C m-2 d-1}

TotRespMegSF = 0.000059*Tlim {mmol C m-2 d-1}

! Stable Isotope Data

13C_Det_w = -27.0 {delta 13C}

13C_Det = -25.9 {delta 13C}

13C_Bac = -26.6 {delta 13C}

13C_ChBac = -20.4 {delta 13C}

13C_MacDF = -24.8 {delta 13C}

13C_MacSF = -27.0 {delta 13C}

13C_MacES = 0.0 {delta 13C}

13C_MacPS = -25.3 {delta 13C}

13C_MegDF = -26.1 {delta 13C}

13C_MegSF = -27.0 {delta 13C}

! Sulphide Flux

minSulphideFlux = 0.08 {mmol C m-2 d-1}

maxSulphideFlux = 7.45 {mmol C m-2 d-1}

! Generic Data

! Detritus

minBur = 0.01 {-}

maxBur = 0.03 {-}

maxRelDOCefflux = 0.10 {-}

! Bacteria

minBGE = 0.05 {-}

maxBGE = 0.45 {-}

minLysBP = 0.30 {-}

maxLysBP = 0.80 {-}

minChemoEff = 0.10 {-}

maxChemoEff = 0.50 {-}

! Macrofauna

minGrowthMac = Tlim*0.01 {d-1}

maxGrowthMac = Tlim*0.05 {d-1}

minMacAss = 0.20 {-} !assimilation efficiency

maxMacAss = 0.75 {-}

minMacNGE = 0.30 {-} !net growth efficiency

maxMacNGE = 0.70 {-}

minMacExcrete = 0.25 {-}

maxMacExcrete = 0.80 {-}

MacRespUpper = 1.50 {-}

MacRespLower = 0.50 {-}

MaintRespMacDF = Tlim*0.001*MacroDepositFeeder {mmol C m-2 d-1}

MaintRespMacSF = Tlim*0.001*MacroSuspensionFeeder {mmol C m-2 d-1}

MaintRespMacPS = Tlim*0.001*MacroPredatorScavenger {mmol C m-2 d-1}

MaintRespMacES = Tlim*0.001*MacroEndosymbiotic {mmol C m-2 d-1}

! Megafauna

minGrowthMeg = Tlim*0.0027 {d-1}

maxGrowthMeg = Tlim*0.014 {d-1}

minMegAss = 0.20 {-} !assimilation efficiency

maxMegAss = 0.75 {-}

minMegExcrete = 0.25 {-}

maxMegExcrete = 0.80 {-}

minMegNGE = 0.50 {-} !net growth efficiency

maxMegNGE = 0.70 {-}

MegRespUpper = 1.50 {-}

MegRespLower = 0.50 {-}

MaintRespMegDF = Tlim*0.00001*MegaDepositFeeder {mmol C m-2 d-1}

MaintRespMegSF = Tlim*0.00001*MegaSuspensionFeeder {mmol C m-2 d-1}

## END PARAMETERS

### VARIABLES

!-- Detritus

OMInput = Det_w -> Det + Det_w -> MacSF + Det_w -> MegSF + ChBacProd + MacESProd

POCdeposition = Det_w -> Det

DetDis = Det -> DOC

BurialOC = Det -> Det_s

! DOC

DOCefflux = DOC -> DOC_w

! Bacteria

BacResp = Bac -> Respiration

BacUpt = DOC -> Bac

BacDocProd = Bac -> DOC

BacProd = BacUpt - BacResp

ChBacResp = ChBac -> Respiration

ChBacUpt = flowto(ChBac)

ChBacDocProd = ChBac -> DOC

ChBacProd = ChBacUpt - ChBacResp

! Macrofauna

MacESUpt = flowto(MacES)

MacESProd = MacESUpt - MacESResp

MacESResp = MacES -> Respiration

MacEsDetProd = MacES -> Det

MacESAss = MacESupt - MacESDetProd

MacESPro = MacESAss - MacESResp

MacDFResp = MacDF -> Respiration

MacDFDetProd = MacDF -> Det

MacDFupt = flowto(MacDF)

MacDFAss = MacDFupt - MacDFDetProd

MacDFPro = MacDFAss - MacDFResp

MacDFGrowth = MacDFAss - MacDFresp + MaintRespMacDF

MacSFResp = MacSF -> Respiration

MacSFDetProd = MacSF -> Det

MacSFupt = flowto(MacSF)

MacSFAss = MacSFupt - MacSFDetProd

MacSFPro = MacSFAss - MacSFResp

MacSFGrowth = MacSFAss - MacSFresp + MaintRespMacSF

MacPSResp = MacPS -> Respiration

MacPSDetProd = MacPS -> Det

MacPSupt = flowto(MacPS)

MacPSAss = MacPSupt - MacPSDetProd

MacPSPro = MacPSAss - MacPSResp

! Megafauna

MegDFResp = MegDF -> Respiration

MegDFDetProd = MegDF -> Det

MegDFupt = flowto(MegDF)

MegDFAss = MegDFupt - MegDFDetProd

MegDFPro = MegDFAss - MegDFResp

MegDFGrowth = MegDFAss - MegDFresp + MaintRespMegDF

MegSFResp = MegSF -> Respiration

MegSFDetProd = MegSF -> Det

MegSFupt = flowto(MegSF)

MegSFAss = MegSFupt - MegSFDetProd

MegSFPro = MegSFAss - MegSFResp

MegSFGrowth = MegSFAss - MegSFresp + MaintRespMegSF

! Totals

BacterialResp = BacResp + ChBacResp

MacResp = MacDFResp + MacSFResp + MacPSResp + MacESResp

MegResp = MegDFResp + MegSFResp

Suspensionfeeding = Det_w -> MacSF + Det_w -> MegSF

NetSuspensionfeeding = Suspensionfeeding - MacSFResp - MegSFResp

TotInSituProd = DIC -> ChBac + DIC -> MacES

NetInSituInput = ChBacProd + MacESPro

MacPredation = MacES -> Predation + MacDF -> Predation + MacSF -> Predation + MacPS -> Predation

MegPredation = MegDF -> Predation + MegSF -> Predation

TotPredation = MacPredation + MegPredation

ExtPredation = flowto(Predation)

TotResp = BacterialResp + MacResp + MegResp

TotSinkingOM = POCdeposition + Suspensionfeeding

NetSinkingOMInput = POCdeposition + NetSuspensionfeeding

TotOMinput = POCdeposition + Suspensionfeeding + TotInSituProd

NetTotOMInput = NetInSituInput + NetSinkingOMInput

TotDetProd = flowto(Det)

#### END VARIABLES

!-------------------------------------------------------------------!

! !

! SYSTEM DECLARATION !

! (STOCKS, EXTERNALS, FLOWS) !

! !

!-------------------------------------------------------------------!

#### STOCK

Det = Detritus

DOC

Bac = HeterotrophicBac

ChBac = FreeChemoautotrophicBac

MacES = MacroEndosymbiotic

MacDF = MacroDepositFeeder

MegDF = MegaDepositFeeder

MacPS = MacroPredatorScavenger

MacSF = MacroSuspensionFeeder

MegSF = MegaSuspensionFeeder

##### END STOCK

##### EXTERNALS

Det_s !Burial

Predation !e.g. Fish/ Megafaunal Predators

Respiration

DIC

DOC_w

Det_w

##### END EXTERNALS

##### FLOW

! Detritus production/ Mortality

Det_w -> Det

DOC -> DOC_w

Det -> DOC

Bac -> DOC

ChBac -> DOC

MacDF -> Det

MacES -> Det

MacSF -> Det

MacPS -> Det

MegDF -> Det

MegSF -> Det

! Bacteria

DIC -> ChBac

DOC -> Bac

! Macrofauna

DIC -> MacES

Det -> MacDF

ChBac -> MacDF

Bac -> MacDF

Det_w -> MacSF

Bac -> MacPS

ChBac -> MacPS

MacES -> MacPS

MacDF -> MacPS

MacSF -> MacPS

! Megafauna

Det -> MegDF

ChBac -> MegDF

Bac -> MegDF

Det_w -> MegSF

! Respiration

Bac -> Respiration

ChBac -> Respiration

MacES -> Respiration

MacDF -> Respiration

MacSF -> Respiration

MacPS -> Respiration

MegDF -> Respiration

MegSF -> Respiration

! Predation

MacES -> Predation

MacDF -> Predation

MacSF -> Predation

MacPS -> Predation

MegDF -> Predation

MegSF -> Predation

! Burial

Det -> Det_s

###### END FLOW

!------------------------------------------------------------------!

! !

! DATA DECLARATION !

! (RATES, EQUATIONS, CONSTRAINTS) !

! !

!------------------------------------------------------------------!

####### RATE

! all rates zero

##### END RATE

##### EQUATION

Flow(Det,MacDF)*13C_Det + Flow(ChBac,MacDF)*13C_ChBac + Flow(Bac,MacDF)*13C_Bac - &

Flow(Det,MacDF)*13C_MacDF - Flow(ChBac,MacDF)*13C_MacDF - Flow(Bac,MacDF)*13C_MacDF = 0

Flow(Det,MegDF)*13C_Det + Flow(ChBac,MegDF)*13C_ChBac + Flow(Bac,MegDF)*13C_Bac - &

Flow(Det,MegDF)*13C_MegDF - Flow(ChBac,MegDF)*13C_MegDF - Flow(Bac,MegDF)*13C_MegDF = 0

Flow(Bac,MacPS)*13C_Bac + Flow(MacSF,MacPS)*13C_MacSF + Flow(ChBac,MacPS)*13C_ChBac + &

Flow(MacES,MacPS)*13C_MacES + Flow(MacDF,MacPS)*13C_MacDF - &

Flow(Bac,MacPS)*13C_MacPS - Flow(MacSF,MacPS)*13C_MacPS - Flow(ChBac,MacPS)*13C_MacPS - &

Flow(MacES,MacPS)*13C_MacPS - Flow(MacDF,MacPS)*13C_MacPS = 0

flowto(MacES) = 0

###### END EQUATION

##### CONSTRAINT

! POC

POCdeposition = [minPOCdeposition,maxPOCdeposition]

! Totals

TotResp = [minTotResp,maxTotResp]

! Bacteria

ChBacUpt = [minChemoEff, maxChemoEff]*[minSulphideFlux, maxSulphideFlux]

ChBacProd = [minBGE,MaxBGE]*ChBacUpt

ChBacDocProd = [minLysBP,maxLysBP]*ChBacProd

BacProd = [minBGE,MaxBGE]*BacUpt

BacDocProd = [minLysBP,maxLysBP]*BacProd

! Macrofauna ES

MacESAss - MacESresp + MaintRespMacES = [minMacNGE, maxMacNGE]*MacESAss

MacESAss - MacESResp + MaintRespMacES = [minGrowthMac, maxGrowthMac]*MacES

! Macrofauna DF

MacDFresp = [MacRespLower, MacRespUpper]*TotRespMacDF

MacDFAss = [minMacAss, maxMacAss]*MacDFUpt

MacDFDetProd = [minMacExcrete, maxMacExcrete]*MacDFUpt

MacDFAss - MacDFresp + MaintRespMacDF = [minMacNGE, maxMacNGE]*MacDFAss

MacDFAss - MacDFResp + MaintRespMacDF = [minGrowthMac, maxGrowthMac]*MacDF

! Macrofauna SF

MacSFresp = [MacRespLower, MacRespUpper]*TotRespMacSF

MacSFAss = [minMacAss, maxMacAss]*MacSFUpt

MacSFDetProd = [minMacExcrete, maxMacExcrete]*MacSFUpt

MacSFAss - MacSFresp + MaintRespMacSF = [minMacNGE, maxMacNGE]*MacSFAss

MacSFAss - MacSFResp + MaintRespMacSF = [minGrowthMac, maxGrowthMac]*MacSF

! Macrofauna PS

MacPSresp = [MacRespLower, MacRespUpper]*TotRespMacPS

MacPSAss = [minMacAss, maxMacAss]*MacPSUpt

MacPSDetProd = [minMacExcrete, maxMacExcrete]*MacPSUpt

MacPSAss - MacPSresp + MaintRespMacPS = [minMacNGE, maxMacNGE]*MacPSAss

MacPSAss - MacPSResp + MaintRespMacPS = [minGrowthMac, maxGrowthMac]*MacPS

! Megafauna DF

MegDFresp = [MegRespLower, MegRespUpper]*TotRespMegDF

MegDFAss = [minMegAss, maxMegAss]*MegDFUpt

MegDFDetProd = [minMegExcrete, maxMegExcrete]*MegDFUpt

MegDFAss - MegDFresp + MaintRespMegDF = [minMegNGE, maxMegNGE]*MegDFAss

MegDFAss - MegDFResp + MaintRespMegDF = [minGrowthMeg, maxGrowthMeg]*MegDF

! Megafauna SF

MegSFresp = [MegRespLower, MegRespUpper]*TotRespMegSF

MegSFAss = [minMegAss, maxMegAss]*MegSFUpt

MegSFDetProd = [minMegExcrete, maxMegExcrete]*MegSFUpt

MegSFAss - MegSFresp + MaintRespMegSF = [minMegNGE, maxMegNGE]*MegSFAss

MegSFAss - MegSFResp + MaintRespMegSF = [minGrowthMeg, maxGrowthMeg]*MegSF

!-- Efflux of DOC from the sediment

DOCefflux < maxRelDOCefflux*TotResp !Burdige GCA 63:1507-1515

! Burial

BurialOC = [minBur, maxBur]*TotOMinput

##### END CONSTRAINT


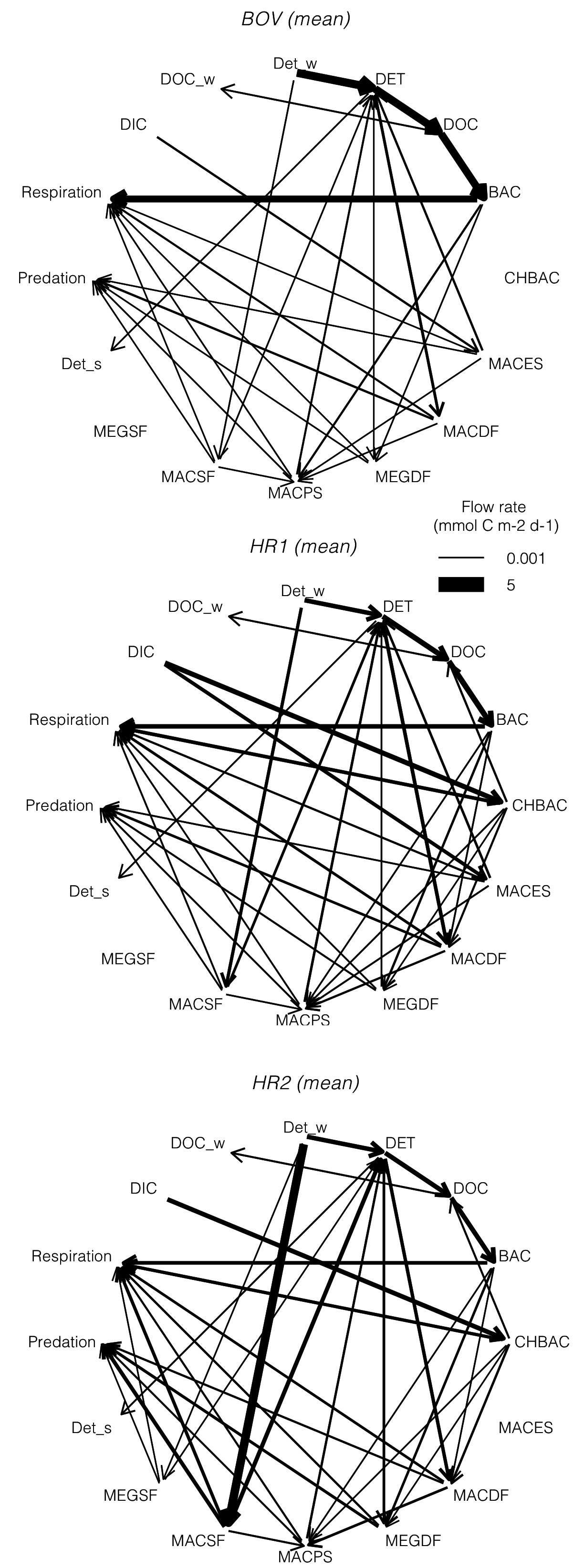
Supplementary Figure 1 – Mean flow rates between compartments at each site.


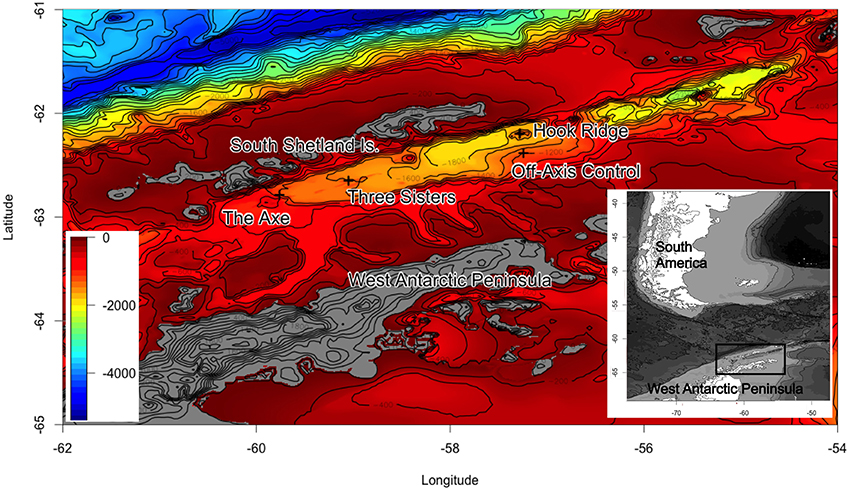


Supplementary Figure 2 – Map of sampling locations in the Bransfield Strait (after Bell, J. B. Woulds, C. Brown, L. E., Little, C. T. S., Sweeting, C. J., Reid, W. D. K., Glover, A. G. Macrofaunal ecology of sedimented hydrothermal vents in the Bransfield Strait, Antarctica. *Frontiers in Marine Science* **3**, 32, DOI:[10.3389/fmars.2016.00032](http://journal.frontiersin.org/article/10.3389/fmars.2016.00032/full) (2016))


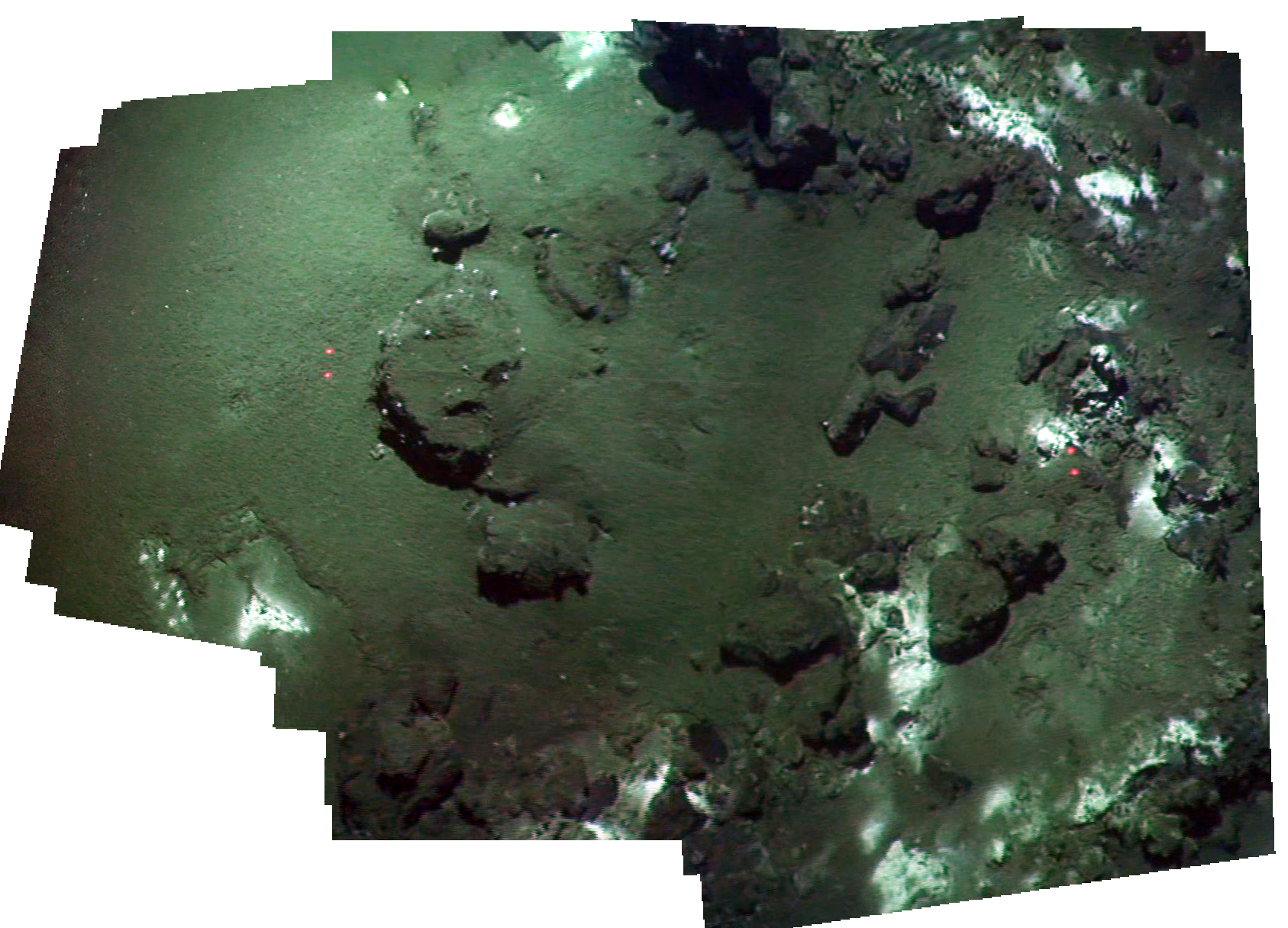


Supplementary Figure 3 – Seafloor imagery mosaic at Hook Ridge 1. Red lasers spaced 10 cm apart.


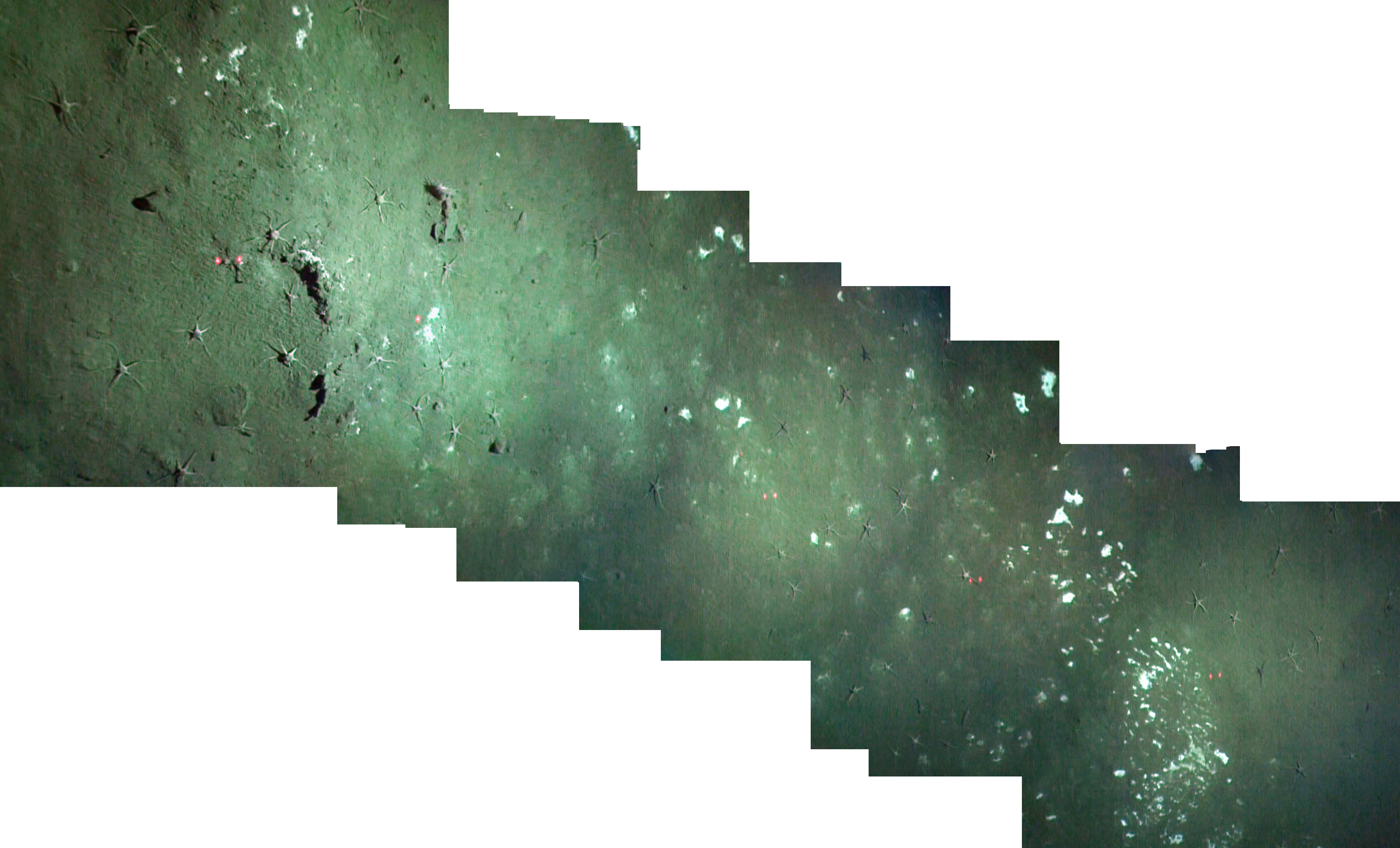


Supplementary Figure 4 – Seafloor imagery mosaic at Hook Ridge 2. Red lasers spaced 10 cm apart.


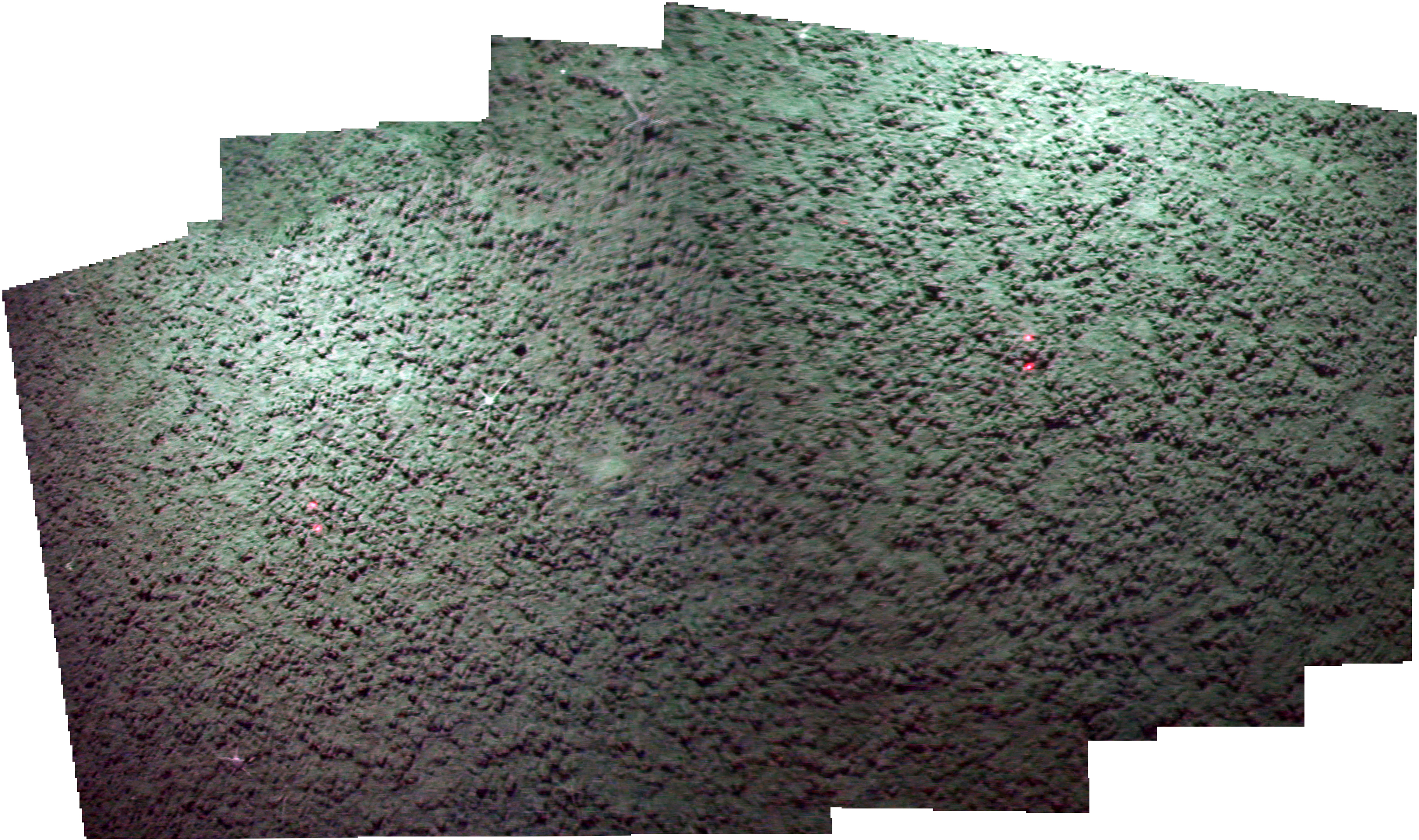


Supplementary Figure 5 – Seafloor imagery mosaic at an off-vent site. N.B. this was not the off-vent site described in this study, for which no imagery is available, but an alternate off-vent site (the Three-Sisters – see Bell, J. B. Woulds, C. Brown, L. E., Little, C. T. S., Sweeting, C. J., Reid, W. D. K., Glover, A. G. Macrofaunal ecology of sedimented hydrothermal vents in the Bransfield Strait, Antarctica. *Frontiers in Marine Science* **3**, 32, DOI:[10.3389/fmars.2016.00032](http://journal.frontiersin.org/article/10.3389/fmars.2016.00032/full) (2016)) that had a similar macrofaunal composition. Red lasers spaced 10 cm apart.
